# Comprehensive microRNA profiling in B-cells of human centenarians by massively parallel sequencing

**DOI:** 10.1186/1471-2164-13-353

**Published:** 2012-07-31

**Authors:** Saurabh Gombar, Hwa Jin Jung, Feng Dong, Brent Calder, Gil Atzmon, Nir Barzilai, Xiao-Li Tian, Joris Pothof, Jan HJ Hoeijmakers, Judith Campisi, Jan Vijg, Yousin Suh

**Affiliations:** 1 Department of Systems and Computational Biology, Albert Einstein College of Medicine, Bronx, NY 10461, USA; 2 Department of Genetics, Albert Einstein College of Medicine, Bronx, NY 10461, USA; 3 Department of Medicine, Albert Einstein College of Medicine, Bronx, NY 10461, USA; 4 Institute for Aging Research, Diabetes Research and Training Center, Albert Einstein College of Medicine, Bronx, NY, 10461, USA; 5 Department of Human Population Genetics, Peking University, Beijing 100871, China; 6 Department of Genetics, Erasmus Medical Center, Rotterdam, The Netherlands; 7 Buck Institute for Research on Aging, Novato, CA 94945, USA; 8 Institute of Aging Research, Guangdong Medical College, Dongguan 523808, China

**Keywords:** MicroRNA, Centenarians, Aging, Life span, Massively parallel sequencing

## Abstract

**Background:**

MicroRNAs (miRNAs) are small, non-coding RNAs that regulate gene expression and play a critical role in development, homeostasis, and disease. Despite their demonstrated roles in age-associated pathologies, little is known about the role of miRNAs in human aging and longevity.

**Results:**

We employed massively parallel sequencing technology to identify miRNAs expressed in B-cells from Ashkenazi Jewish centenarians, i.e., those living to a hundred and a human model of exceptional longevity, and younger controls without a family history of longevity. With data from 26.7 million reads comprising 9.4 × 10^8^ bp from 3 centenarian and 3 control individuals, we discovered a total of 276 known miRNAs and 8 unknown miRNAs ranging several orders of magnitude in expression levels, a typical characteristics of saturated miRNA-sequencing. A total of 22 miRNAs were found to be significantly upregulated, with only 2 miRNAs downregulated, in centenarians as compared to controls. Gene Ontology analysis of the predicted and validated targets of the 24 differentially expressed miRNAs indicated enrichment of functional pathways involved in cell metabolism, cell cycle, cell signaling, and cell differentiation. A cross sectional expression analysis of the differentially expressed miRNAs in B-cells from Ashkenazi Jewish individuals between the 50^th^ and 100^th^ years of age indicated that expression levels of miR-363* declined significantly with age. Centenarians, however, maintained the youthful expression level. This result suggests that miR-363* may be a candidate longevity-associated miRNA.

**Conclusion:**

Our comprehensive miRNA data provide a resource for further studies to identify genetic pathways associated with aging and longevity in humans.

## Background

MicroRNAs (miRNAs) are small non-coding RNAs that regulate gene expression at the post-transcriptional level [1]. Mature miRNAs, between 18–25 bp in length, are transcribed as primary-miRNA (pri-miRNA) molecules which contain a characteristic stem loop structure. This stem loop targets pri-miRNA for processing by a number of RNAses, namely Drosha and Dicer, which produce a short RNA duplex [1]. From the duplex one or both strands is incorporated into the RNA inducing silencing complex (RISC), resulting in an active miRNA. Active miRNAs target the 3^′^ UTR of a mRNA based on sequence homology [2]. The nucleotides in the 2–7 position of the 5^′^ end of the mature miRNA comprise a “seed region”. Once an mRNA is targeted by a miRNA it can be degraded or its translation can be repressed through conserved mechanisms leading to downregulation of gene expression [1,3].

First described in *C. elegans*[3], many miRNAs have been discovered across a wide range of species, including humans. There is increasing evidence that miRNAs are critical in a number of essential biological processes, including cell differentiation [4], immune response [5], cancer [6,7], and life span [8]. Thus far, 1048 human miRNA sequences have been identified through cloning, sequencing, or computational analysis (mirBase, release 16, 2010) [9-11].

The multitude of important roles played by miRNAs suggests that they are a critical component of gene regulatory networks. However, the quantification of miRNAs has been technically challenging due to their small size, low copy number, interference from other small RNAs, and contamination by degradation products of mRNAs or other RNA species. Until recently only known and computationally predicted miRNAs have been interrogated using hybridization-based array methods, which suffer from cross-hybridization, and inability to discover novel miRNAs due to limited array content. The increased availability and affordability of massively parallel sequencing now offer an opportunity to gain a high-resolution view of miRNA expression, overcoming past experimental limitations [12]. MiRNA-seq has been utilized to discover novel and quantify expression levels of miRNAs in several species, including humans [13,14].

Expression levels of genes are heritable in humans as quantitative phenotypes, measurable in a variety of cell types, including B-cells [15]. Recent studies, including our own [16,17], have demonstrated that B cells reflect functional characteristics of the donor and can be a useful tool for studying genotype-driven molecular endpoints such as gene expression, and expression quantitative trait locus (eQTL) analysis [18,19]. B cells can act as surrogate tissues whenever there is correlation between the expression levels of B cells and phenotypes of interest [20,21] and a large number of eQTLs originally identified in B cells can also be detected in multiple primary tissues [22,23]. Thus B-cells have been increasingly utilized for expression quantitative trait loci (eQTLs) studies [19,24] and as a cell model to assess gene expression responses [25]. While comprehensive mRNA expression data for human B-cells, obtained by RNA-seq, are available for this purpose [26], to date no such analysis was performed to identify miRNAs expressed in B-cells. Here, we present comprehensive miRNA transcriptome profiles of B-cells from Ashkenazi Jewish centenarians and younger control individuals by miRNA-seq, providing a resource that could serve as a basis for establishing gene regulatory interactions between miRNAs and their target mRNAs in human B-cells.

## Results

### Discovery of miRNAs expressed in B-cells of centenarians and controls

**Figure 1:**
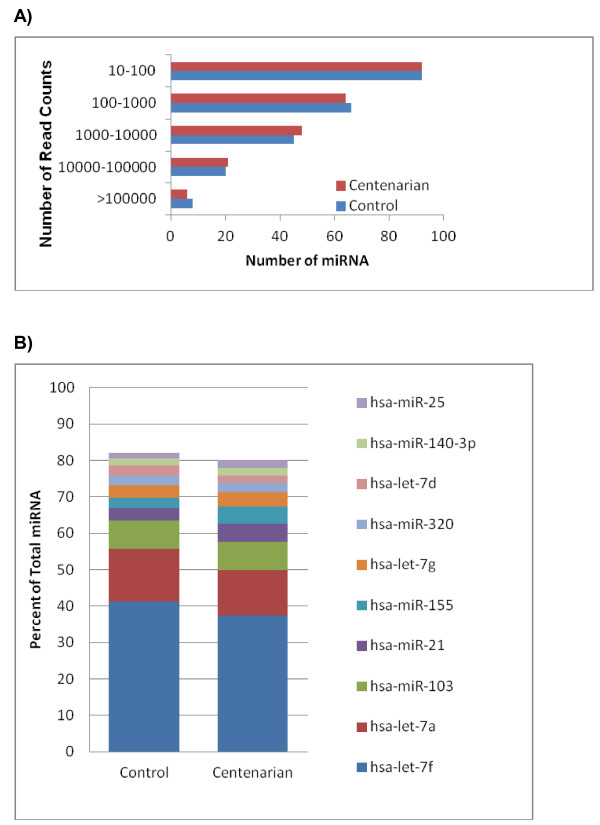
**Known miRNAs discovered in B-cells from centenarians and controls.** (**A**) Distribution of the miRNAs expressed in B-cells according to their sequence counts in centenarians and controls. On the vertical axis is a bin for miRNAs with sequence read counts within the given range. In the horizontal axis is the numbers of miRNAs found for each of the magnitudes of expression. (**B**) Top 10 known miRNAs expressed in B-cells from centenarians and controls, which constitute nearly 80% of all miRNAs expressed in the B-cells.

We generated small RNA libraries of B-cells from 3 Ashkenazi Jewish female centenarians (mean age 104) and 3 Ashkenazi Jewish female younger controls (mean age 63). Sequencing of these libraries by an Illumina technology platform yielded a total of 12.9 × 10^6^ reads from centenarians and 13.8 × 10^6^ reads from controls (Additional file 1: Table S1). After removal of low quality reads and redundancy, we had a total of 1.1 × 10^6^ and 1.0 × 10^6^ unique reads for the centenarians and the controls, respectively. Of the unique reads, those with a read number greater than 10 in more than 50% (n = 3) of the individuals sequenced (n = 6) were aligned to a database of known miRNAs ( http://www.mirbase.org/) and other known small RNA species. MiRNAs with less than 10 reads were not included due to the error rate of Illumina sequencing and stochastic variation in gene expression [12]. For all comparisons, the number of reads for a given miRNA was normalized by division of the total number of miRNA reads in that library, yielding a normalized read count for each miRNA [12]. We identified a total of 276 known miRNAs (Additional file 2: Table S2) with a wide dynamic range of read counts ranging from 10 to over 1 million (Figure 1A). About 11% of these miRNAs had a copy number greater than 10,000 and about 67% a copy number less than 1,000. The identification of 276 miRNAs out of the 1048 reported human miRNAs (mirBase, release 16, 2010) [9-11] is in the similar range as observed by others for a particular cell type [14,27], suggesting a saturation of miRNA-sequencing. The normalized read count for each miRNA indicated that let-7f was the most abundant miRNA detected in B-cells (Figure 1B). The top 10 miRNAs by read count corresponded to ~80% of all sequence reads in the B-cells (Figure 1B). A complete list of miRNAs sequenced in each library, normalized read counts, and fold differences between centenarian and control cells is provided in Additional file 2: Table S2.

To discover previously unknown novel miRNAs among our short sequence RNAs that did not align to the known miRNA database, we took advantage of the fact that miRNAs are sequentially processed to their mature form from stem-loop containing pre-miRNAs. Utilizing the mirDeep program [28] we identified small RNAs that correspond to processing of characteristic stem loop containing pre-miRNAs. MirDeep uses a number of criteria to select novel miRNAs [29], including a minimum free energy of less than −25 kcal/mol, 25% sequence homology with known miRNA precursors, sufficient GC content, and no alignment to too many different genomic locations. We discovered a total of 8 potentially novel miRNAs with a characteristic stem loop structure that meet these criteria (Table 1). None of the novel miRNAs were found to have expression over 100,000 reads in any of our samples. All putative miRNA precursors, with the exception of sRNA8, had a lowest free energy and best folding structure resembling known miRNA (Table 1). All of the putative miRNAs and their genomic locations have been submitted to mirBase.

**Table 1:** Novel miRNAs discovered in B-cells, their chromosome location, mature sequence, and putative folding structure

**Temporary name**	**Genomic location**	**Mature sequence**	**Fold**
sRNA1	chr11:95714237–95714346 || chr11:41380469- 41380478	ATCCCACCACTGCCACCA	
sRNA2	chr11:46193225-46193334	TATCCCGGACGAGCCCCCA	
sRNA3	chr1:218470812-218470921	CATGTGGGCTAGTTTCAGACAGGT	
sRNA4	chr15:81221792–81221896 || chr15:545116- 545175	AAGGTAGATAGAACAGGTCTTG	
sRNA5	chr17:76874327-76874436	ACCTTGGCTCTAGACTGCTTACT	
sRNA6	chr17:24191370-24191479	GCTCTGACTTTATTGCACTACT	
sRNA7	chr9:78376641-78376750	CGCTAAACCATTCGTAGACGACCTGCTTC	
sRNA8	chr11:121147952–121148061 || chr11:13287835- 13287776	GGCTGGTCCGAAGGTAGTGAGT	

**Base-pair probabilities:**

Novel miRNA were predicted using the mirDeep package. The pre-miRNA sequence of sRNA1 and sRNA5 were present in two genomic locations where miRNA have not been previously predicted. The fold column was generated from mFold [30].

### IsomiRs and variability of miRNA processing

Mature miRNAs are not always produced with the same sequence even when originating from the same precursor miRNA. Such mature variants have been termed “isomiRs” [13]. A tissue-specific expression pattern of isomiRs during development has been demonstrated in Drosophila, suggesting a specific role of isomiRs in gene regulatory networks [31]. A failure to identify all isomiRs can alter sequence counts and lead to incorrect conclusions of miRNA abundance [13]. Most of the variation in isomiRs can be described by the variability in Dicer 1 and Drosha processing of the miRNAs [32]. We compared the composition of isomiRs between B-cells originating from centenarians vs. controls and identified a total of 694 variants from 261 miRNAs. Similar isomiR patterns were identified in centenarian and control cells, suggesting that global miRNA biogenesis is not significantly different between centenarians and controls. A subset of identified isomiRs and their compositions within the groups is listed in Table 2. We found variants within our most abundant miRNAs (e.g., hsa-let-7a and hsa-miR-21) and within our differentially expressed miRNAs (e.g., hsa-miR148a and hsa-miR-193b). There is evidence that the most abundant isomiR in a sample is not always the same as the reference sequence reported in mirBase [33]. Indeed in our B-cells a variant of hsa-miR-21 not reported in mirBase was the dominant isomiR. A complete list of sequenced isomiRs from each individual miRNA discovered in B-cells is available in Additional file 3: Table S3.

**Table 2:** Representative IsomiRs detected in B-cells

***miRNA***	***Sequence***	***% Control***	***% Centenarian***
hsa-miR-21	TAGCTTATCAGACTGATGTTGA**C**	53.8%	52.0%
	**TAGCTTATCAGACTGATGTTGA**	43.3%	44.9%
	TAGCTTATCAGACTGATGTTGA**A**	2.7%	2.8%
	TAGCTTATCAGACTGATGTTGA**T**	0.2%	0.3%
hsa-miR-193b	**AACTGGCCCTCAAAGTCCCGCT**	84.8%	88.6%
	AACTGGCCCTCAAAGTCCCGCT**T**	8.7%	5.9%
	AACTGGCCCTCAAAGTCCCGCT**A**	6.2%	5.2%
	AACTGGCCCTCAAAGTCCCGCT**G**	0.3%	0.3%
hsa-let-7a	**TGAGGTAGTAGGTTGTATAGTT**	86.8%	86.9%
	TGAGGTAGTAGGTTGTATAGTT**A**	9.3%	9.2%
	TGAGGTAGTAGGTTGTATAGTT**T**	3.6%	3.5%
	TGAGGTAGTAGGTTGTATAGTT**G**	0.4%	0.3%
hsa-miR-148a	**TCAGTGCACTACAGAACTTTGT**	94.3%	94.3%
	TCAGTGCACTACAGAACTTTGT**C**	3.9%	3.6%
	TCAGTGCACTACAGAACTTTGT**T**	1.3%	1.4%
	TCAGTGCACTACAGAACTTTGT**A**	0.5%	0.7%

Some of the top isomiRs are shown with the percent contribution of each isomiR to the total read count within each group. Reference sequence is denoted in bold and isomer variation from the reference is noted with bold and underscore.

### MiRNAs differentially expressed between centenarians and controls

**Figure 2:**
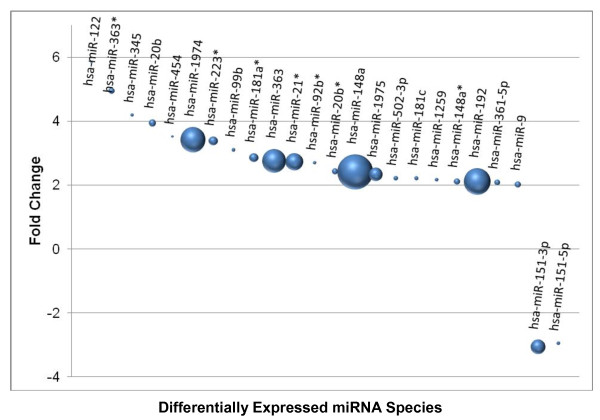
**Relative abundance and fold changes of miRNAs differentially expressed between centenarians and controls.** Each sphere along the X-axis represents a single miRNA that was found differentially expressed between centenarians and controls. The diameter of a sphere represents the relative abundance of the miRNA read count. The Y-axis denotes the fold changes between the two groups. Positive values denote up-regulation, and negative values denote down-regulation, of the miRNAs in centenarians as compared to controls.

To identify differentially expressed miRNAs, we performed a Fisher’s exact test and adjusted for multiple testing with Bonferroni correction [33-35]. We identified a total of 24 differentially expressed known miRNAs with a fold change > 2.0 and Bonferroni corrected p-value < 0.05 (Table 3). Of the 24 differentially expressed miRNAs, 22 were upregulated and only two downregulated in centenarians as compared to controls (Figure 2). Normalized read counts for the differentially expressed miRNAs ranged from the 20s to over 100,000. A complete list of miRNAs sequenced in each library, normalized read counts, and fold changes between centenarians and controls is provided in Additional file 2: Table S2.

**Table 3:** Differentially expressed miRNAs in B-cells from centenarians vs. controls

**miRNA**	**Up regulated in**	**Control counts (standard error)**	**Centenarian counts (standard error)**	**P values**	**Bonferonni adjusted p values**	**Fold change**
hsa-miR-122	Centenarian	19 (6.26)	102 (6.39)	1.06E-16	2.44E-14	5.83
hsa-miR-363*	Centenarian	184 (23.30)	837 (81.32)	3.6E-112	8.2E-110	4.94
hsa-miR-345	Centenarian	59 (10.65)	227 (32.69)	9.84E-28	2.27E-25	4.18
hsa-miR-20b	Centenarian	306 (25.72)	1108 (240.38)	7.7E-122	1.8E-119	3.93
hsa-miR-454	Centenarian	39 (6.55)	126 (34.59)	1.02E-13	2.36E-11	3.51
hsa-miR-1974	Centenarian	4515 (660.27)	14179 (668.31)	0	0	3.41
hsa-miR-223*	Centenarian	580 (39.17)	1800 (204.29)	2.2E-167	5.1E-165	3.37
hsa-miR-99b	Centenarian	97 (15.32)	276 (83.78)	2.15E-24	4.96E-22	3.09
hsa-miR-181a*	Centenarian	627 (24.30)	1641 (307.63)	4.5E-123	1E-120	2.84
hsa-miR-363	Centenarian	4732 (260.52)	11971 (2286.46)	0	0	2.75
hsa-miR-21*	Centenarian	2529 (286.89)	6313 (1153.62)	0	0	2.71
hsa-miR-92b*	Centenarian	99 (11.67)	245 (42.00)	2.45E-18	5.66E-16	2.69
hsa-miR-20b*	Centenarian	319 (77.91)	708 (84.88)	5.78E-42	1.33E-39	2.41
hsa-miR-148a	Centenarian	11599 (1225.92)	25583 (655.62)	0	0	2.40
hsa-miR-1975	Centenarian	1654 (86.54)	3529 (335.65)	2.1E-188	4.9E-186	2.32
hsa-miR-502-3p	Centenarian	191 (13.48)	387 (14.66)	4.03E-20	9.31E-18	2.20
hsa-miR-181c	Centenarian	153 (6.46)	310 (63.42)	1.8E-16	4.15E-14	2.20
hsa-miR-1259	Centenarian	106 (25.78)	210 (29.26)	4.01E-11	9.27E-09	2.15
hsa-miR-148a*	Centenarian	339 (46.74)	656 (14.26)	3.38E-30	7.82E-28	2.10
hsa-miR-192	Centenarian	7126 (1026.34)	13754 (2446.01)	0	0	2.10
hsa-miR-361-5p	Centenarian	307 (20.35)	587 (89.41)	1.65E-26	3.81E-24	2.08
hsa-miR-9	Centenarian	341 (26.37)	629 (37.72)	3.8E-26	8.78E-24	2.00
hsa-miR-151-3p	Control	4515 (1288.82)	1352 (223.77)	0	0	3.07
hsa-miR-151-5p	Control	257 (72.35)	80 (13.37)	7.22E-20	1.67E-17	2.96

All miRNA determined to be differentially expressed with a Bonferroni adjusted p-value < 0.05 and fold change > 2.0.

### Cross platform comparison of differential miRNA expression

**Figure 3:**
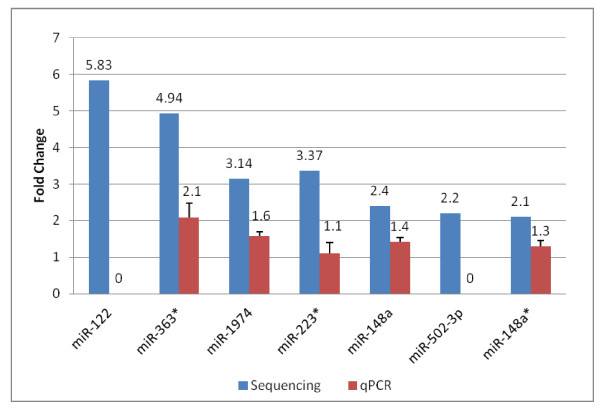
**Cross platform comparison of miRNA differential expression.** Quantitative RT-PCR with TaqMan probes was used to validate differential expression of miRNAs detected by miRNA-seq. Standard error of the mean for qPCR values are shown.

Until recently, differentially expressed miRNAs were discovered using miRNA microarray technology, which is subject to certain limitations such as difficulties in detecting low abundance miRNAs and a limited dynamic range, essentially constraining the detection of subtle fold changes. These limitations call for an additional technical validation step, for example, quantitative real time PCR (qRT-PCR), which has been considered the gold-standard of differential analysis of gene expression. We conducted qRT-PCR analysis to validate the differential expression of 7 miRNAs out of the 24 miRNAs found to be significantly different in B-cells from centenarians as compared to controls by Illumina sequencing analysis. Five miRNAs (hsa-miR-363*, hsa-miR-1974, hsa-miR-223*, hsa-miR-148a, hsa-miR-148a*) produced expression patterns consistent with the Illumina sequencing data (Figure 3). However, the qRT-PCR data clearly underestimated the fold changes in gene expression when compared with the Illumina sequencing analysis. In addition, qRT-PCR failed to detect the expression of hsa-miR-122 and hsa-miR-502-3p. These results indicate that differential analysis of miRNAs by deep sequencing should be the gold standard for quantification of miRNA expression, especially since there is evidence for both 5^′^ and 3^′^ editing of mature miRNAs, which reduces the efficiency of miRNA detection by hybridization-based method such as qRT-PCR [36].

### Targets of differentially expressed miRNAs

Among the 24 differentially expressed miRNAs, several have been previously characterized with known validated targets (Additional file 4: Table S4), including hsa-miR-148 and hsa-miR-122 [5,37,38]. Target sites of the 24 differentially expressed miRNAs were predicted using available computational approaches, in particular TargetScan ( http://www.targetscan.org) [39,40], miRanda ( http://www.microrna.org/microrna/home.do) [41], and PicTar ( http://pictar.mdc-berlin.de/) [42]. These software packages work by finding an absolute base pair homology of the miRNA seed region (bases 2–8 on the 5^′^ end of the miRNA) to the 3^′^ UTR of all mRNAs. In addition, they take into consideration evolutionary conservation of 3^′^ UTR bases as well as RNA accessibility to the RISC complex. Due to the large number of predicted targets we only considered target genes with 2 predicted sites, an accessible 3^′^UTR and consensus amongst all prediction algorithms [27,43]. Based on this approach we identified predicted target genes for 10 out of the 24 differentially expressed miRNAs (Additional file 4: Table S4). To explore the potential pathways regulated by these miRNAs, we conducted Gene Ontology analysis utilizing the “GoStat” analysis tool ( http://gostat.wehi.edu.au/). We took the total list of predicted and validated targets of the differentially expressed miRNAs and determined if any Gene Ontology categories were over-represented within our list [44]. We found enrichment of functional pathways implicated in the aging process, including cell metabolism, cell cycle, cell signaling, and cell differentiation (Table 4) among other significant GO categories (Additional file 5: Table S5). In addition, we compared our putative target list to the GenAge database which contains a collection of genes implicated in aging [45] using a standard gene set enrichment approach [46]. We found that our target list has a significant over-representation of these genes (Table 4).

**Table 4:** Representative GO categories enriched in targets of differentially expressed miRNAs

**GO TERM**	**GO ID**	**p-Value (Bonferonni Adjusted)**
cell differentiation	GO:0030154	1.52 × 10^-9^
cellular metabolic process	GO:0044237	3.82 × 10^-6^
cell-cell signaling	GO:0007267	1.05 × 10^-5^
cell proliferation	GO:0008283	6.66 × 10^-5^
cell cycle	GO:0007049	8.02 × 10^-4^
GenAge Database		4.5 × 10^-2^

### Cross-sectional analysis of miRNA expression in different age groups

**Figure 4:**
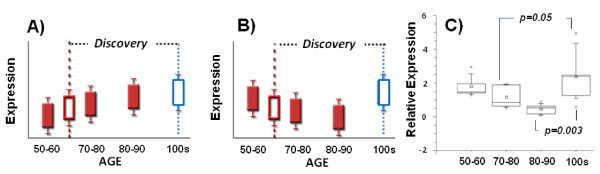
**Cross-sectional expression of select miRNA in different age groups.** The dotted line shows the age of the discovery group (mean age 63) for differential analysis with centenarians (mean age 104). **A**) If differential expression is simply age-related, expression will increase with age. **B**) If the differential expression is longevity-related, patterns of youthful expression will be preserved in centenarians while its expression levels in control individuals decline with age. **C**) A cross-sectional expression analysis of hsa-miR-363* by quantitative RT-PCR showed the youthful preservation mode: its expression levels significantly decline with age in control individuals (p < 0.05), while centenarians maintain the expression levels of the youngest age group (50–60). The significance of expression differences was assessed by student’s t-test. Each age group had 5 individuals while centenarians had 12 individuals.

Since 22 miRNAs out of 24 differentially expressed miRNAs were discovered to be upregulated in centenarians (mean age: 103) as compared to controls (mean age: 63), we set out to determine the mode of differential expression over a range of age groups. If upregulation is simply age-related, expression will increase with age (Figure 4A). In contrast, if the upregulation is longevity-related, patterns of youthful expression will be preserved in centenarians while its expression levels in control individuals decline with age (Figure 4B). Among the differentially expressed miRNAs, we chose hsa-miR-363* for a further validation study because its upregulation in centenarians was detectable by qRT-PCR analysis with > 2 fold change (Figure 3). We performed a cross-sectional study to measure miRNA expression levels in B-cells established from 4 age groups of Ashkenazi Jewish individuals; 50–60 (n = 5), 70–80 (n = 5), 80–90 (n = 5), >100 years (n = 12). We found that the expression levels of hsa-miR-363* significantly declined with age in control individuals (p < 0.05), while centenarians maintained comparable “high” expression levels of the youngest age group (Figure 4C). The expression differences between centenarians and younger elderly controls were significant (p = 0.05 for 70–80 vs. > 100; p = 0.003 for 80–90 vs. > 100) (Figure 4C), suggesting that hsa-miR-363* is a candidate longevity-associated miRNA.

## Discussion

We employed a massively parallel sequencing technology to identify miRNAs expressed in B-cells established from Ashkenazi Jewish Centenarians and younger elderly controls without a family history of longevity. In this study, which represents the first comprehensive studies to analyze miRNAs in human B-cells, we obtained 26.7 million reads comprising 9.4 × 10^8^ bp from 3 centenarian and 3 control individuals. We found a total of 284 miRNAs expressed in human B-cells, eight of which were previously unknown, putative novel miRNAs.

Profiling miRNA transcriptomes has gained importance with increasing evidence for the role of miRNA expression in defining cellular phenotypes. Prior to the advent of increasingly cost-effective, small RNA sequencing, microarrays were the prevailing methods for miRNA expression analysis. However, apart from noise due to cross-hybridization to probes with similar short-sequences, array technologies are limited in several key ways. First, microarrays are limited to the probe sets, eliminating the possibility to discover novel miRNAs. Second, arrays are only able to identify the relative abundance of miRNAs but not their absolute numbers [12]. Sequencing allows accurate detection of expression levels over a wide dynamic range, including low copy number miRNAs and subtle fold changes between test and control groups. Finally, array-based methods cannot differentiate isomiRs (mature miRNAs originated from the same precursor but differing in one or more bases) resulting from the high variability in miRNA processing [13,31]. Using miRNA-seq of human B-cells, we were able to identify novel miRNAs and isomiRs, and detected expression of low-copy miRNAs and subtle fold changes between centenarians and controls, which could not be measured using TaqMan qPCR.

Genome-wide transcriptional profiles of miRNAs in C. elegans [47] and human blood cells [48] showed that the majority of miRNAs decreased in expression with age. Likewise, cellular senescence was accompanied by significant down-regulation in miRNA expression [49]. Our results suggest that centenarians may be more resistant to such age-related down-regulation of miRNAs because the overwhelming majority of significant miRNAs are increased in centenarians as compared to controls in B-cells: we found 22 miRNAs significantly upregulated, with only 2 downregulated, in centenarians as compared to younger controls. Strikingly, expression levels of 5 miRNAs shown to be increased in centenarians (hsa-miR-148a, hsa-miR-345, hsa-miR-361-5p, hsa-miR-192, hsa-miR-454) have been demonstrated to be down-regulated during cellular senescence (Table 5). Among these, hsa-miR-148a was also found to be down-regulated with age in peripheral blood mononuclear cells (PBMCs) [48]. Together, these results suggest that miRNAs associated with longevity can be detected by cross-sectional expression analysis as utilized in this work.

**Table 5:** Relevance of miRNAs differentially expressed in centenarians to senescence and aging

**miRNA**	**Up regulated in centenarians**	**Down regulated in centenarians**	Up regulated in senescence^*^	Down regulated in senescence^*^	Down regulated in aged PBMC^**^
hsa-miR-148a	·			·	·
hsa-miR-345	·			·	
hsa-miR-361-5p	·			·	
hsa-miR-192	·			·	
hsa-miR-454	·			·	
hsa-miR-122	·		·		
hsa-miR-502-3p	·		·		
hsa-miR-151-5p		·	·		

* Determined as differentially expressed in [50].

**Determined as differentially expressed in [48].

By conducting a cross-sectional analysis of miRNA expression in different age groups, we found that expression of hsa-miR-363* significantly declined with age in control individuals, whereas centenarians maintain comparably high expression levels, similar to the observed in the middle age group. The results suggest that hsa-miR-363* is a candidate longevity-associated miRNA. Previously miRNA* sequences were thought to be degraded. However a growing body of work challenges the dogma that miRNA* is simply a non-functional byproduct of miRNA biogenesis, suggesting instead that miRNA* plays a significant role in cellular function and human disease [51]. Furthermore, they are implicated in the aging of C. elegans [52]. The predicted targets of hsa-miR-363* include PTEN, BCL2, AKT1, and IGFBP5 among genes listed in the GenAge database [45]. To our knowledge this is the first cross-sectional analysis of miRNA in a human longevity cohort, identifying a potential longevity-associated miRNA.

**Figure 5:**
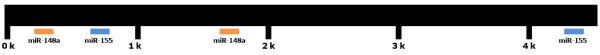
**3**^′^**UTR of Rictor showing multiple miRNA target locations.**

Targets for our differentially expressed miRNAs were obtained through the literature and by bioinformatics means (Additional file 4: Table S4). The predicted and validated targets of the 24 differentially expressed miRNAs are enriched in biological pathways implicated in the aging process, including cell differentiation, cell cycle, cell signaling, and cell metabolism (Table 4), as well as the GenAge database [45]. Of particular interest were the genes involved in cognitive aging (ADAMTS5, APPL1, IGF2, LRP1b), aging-related signaling/transcription (E2F2, FGF7, MLL, MYB , KLF3, PTEN, RICTOR, SMAD2, SOCS5, TCF4), cell cycle/senescence/apoptosis (BCL2L11, CBL , CDC2L6, E2F2, EEF2K), and overlap with the GenAge database of known aging related genes (SHC1, E2F1, STAT3, IGF1, IRS2, PTEN, RPA1, BCL2, SP1, HBP1, MXD1). We searched for potential target genes that were predicted to bind multiple miRNAs found to be differentially expressed in our study because it has been shown that miRNAs can act in an additive manner and the co-targeting by multiple miRNAs would greatly reduce mRNA expression even if the miRNAs were in low copy number [53]. We found that RICTOR (rapamycin-insenstive companion of mTOR) was predicted to bind at least two miRNAs upregulated in centenarians as compared to controls. The 3^′^ UTR of RICTOR and the binding sites for miR-148a and miR-155 (fold change > 1.5) are shown in Figure 5. RICTOR is part of the TOR family of genes which are integral to growth and proliferation, and down-regulation of this pathway is shown to extend lifespan [54].

Longevity is known to have a genetic component in humans. While the heritability of average life expectancy has been estimated to be only ~25% [55,56], studies of centenarians indicate much stronger heritability at old age. For example, siblings of centenarians have a 4 times greater probability of surviving to age 90 than siblings of people with average life span [57]. Achieving a lifespan of 100 years is 17 and 8 times more likely for male or female siblings of centenarians, respectively, compared to their birth cohort [58]. These findings firmly established the utility of human centenarians as a model system to unravel the genetics of longevity. For this study, we used our unique cohort of centenarians and elderly controls without family history of exceptional longevity, all of genetically homogeneous Ashkenazi Jewish descent. This cohort has been used to successfully discover longevity associated genotypes and phenotypes in the past [16,59].

Recently, miRNAs have emerged as critical regulators of gene expression and a link between multiple miRNAs and longevity [8,60] and aging [47] has been demonstrated in *C. elegans*, implicating their role in regulation of lifespan and in the aging process. Since a significant number of miRNAs are evolutionarily conserved [61,62], it is conceivable that miRNAs play a role in human longevity as well. Our finding that most of the differentially expressed miRNAs were upregulated in centenarians could point towards increased resilience of centenarians against an age-related decline in gene regulatory control. By conducting a cross-sectional expression analysis, we found a candidate longevity-associated miRNA, hsa-miR-363* (Figure 4). Hsa-miR-363* shows reduced expression in control individuals with advancing age while maintaining relatively high expression levels in centenarians. Maintenance of youthful expression patterns may be beneficial and longevity-associated miRNAs may confer robustness to gene expression networks, protecting them against age-related deterioration.

In the field of human genetics, most of the surveys of gene expression have been conducted in B-cells because they are readily available and can be used multiple times under controlled experimental conditions [15,63]. B cells act as surrogate tissues whenever there is a correlation between expression levels in these cells and phenotypes of interest [20,21]. However, caution needs to be taken in interpreting the results, especially with negative data, as truly tissue-specific genes will not be detected in B-cells. The lack of a correlation can never be used to infer that the miRNA/gene is not involved in human longevity, and only positive results should be interpreted as in most large-scale discovery-based science.

## Conclusions

Massively parallel sequencing technology allowed us to accurately detect miRNAs expressed in B-cells. Considering the increasing use of B-cells for genetic and functional studies [16,64] our data provides a resource for designing gene expression studies and to study gene regulatory networks mediated by miRNAs. Furthermore, our results from B-cells established from a human longevity cohort may generate an opportunity to explore the possible role of miRNAs in human aging and longevity and to identify genes and pathways that are targets for age-related alteration.

## Methods

### Population and sample collection

All individuals are enrolled in the Longevity Genes Project, and were recruited as described previously [65]. Informed written consent was obtained in accordance with the policy of the Committee on Clinical Investigations of the Albert Einstein College of Medicine. All blood samples were processed at the General Clinical Research Center at the Albert Einstein College of Medicine in order to produce EBV transformed immortalized B-cells as a source of RNA. Total RNA was extracted from immortalized B-cell lines established from centenarians and controls using TRIZOL reagent as recommended by the manufacturer (Invitrogen).

#### Ethics

Experimental research reported in this manuscript has been performed with the approval of the Committee on Clinical Investigations of the Albert Einstein College of Medicine. Research carried out in this manuscript is in compliance with the Helsinki Declaration ( http://www.wma.net/e/policy/b3.htm).

### MiRNA-sequencing

MiRNA-seq was performed as recommended by the manufacturer (Illumina small RNA prep kit v 1.5). 10 μg of total RNA from each sample was resolved on a 15% TBE-Urea polyacrylamide gel followed by the excision of gels corresponding to the 17–35 nucleotides. Small RNAs were isolated from the gel in 300 μl of 0.3 M NaCl for 4 hours at room temperature. The small RNAs were ligated with a biotinylated RNA-DNA 3^′^-adaptor, gel-purified, and ligated with a 5^′^-adaptor. Products with both adaptors were gel-purified, reverse-transcribed, and PCR amplified for 14 cycles. Sequencing was performed on an Illumina GA1 analyzer.

### Data analysis

The sequencing data was provided from the GA1 sequencer in a standard fastq format [66]. The fastq files were trimmed of adapter sequences and of low quality reads (reads which had more than 3 base-calls below sufficient quality value), through a c++ program. These sequences were then collapsed to remove redundancy using the Galaxy Genome Browser tool fastx [67]. At this point sequences from each of the samples was aligned to the known human miRNA/small RNA database or put into the mirDeep pipeline for the discovery of novel miRNAs [28]. To normalize the samples we determined how frequently the miRNA was annotated per the number of reads reported from the sequencer [12]. The basis of this idea is that for a given number of small sequences isolated from a cell there should be on average the same number of total miRNAs from the sample. Read counts from different libraries were normalized to the total reads in each sample [12].

### Statistical analysis

After normalization any reads observed in fewer than 3 samples or with a copy number less than 10 were not considered for analysis; this correction removed extremely low abundance miRNAs. To identify differentially expressed miRNAs, the data was analyzed through Fisher’s exact test using a Bonferonni correction [33-35] for multiple hypothesis testing. Those miRNAs meeting a corrected cutoff with a p-value below 0.05 and with a fold change greater than 2.0 were considered differentially expressed.

### Quantitative RT- PCR analysis

Total RNA was isolated from B-cells using RNA isolation kit (Qiagen, Valencia, CA) and then converted to complementary DNA using TaqMan Reverse Transcription kit (Applied Biosystems, Foster City, CA) with microRNA specific RT primer (Applied Biosystems). A TaqMan® microRNA assay was performed using AB StepOne^TM^ real-time PCR system to quantify relative miRNAs expression in these samples. The 20 μl total volume final reaction mixture consisted of 1 μl of TaqMan microRNA specific primer, 10 μl of 2x Universal Master Mix with no AmpErase® UNG (Applied Biosystems) and 1.3 μl of complementary DNA. PCR was performed using the following conditions: 50°C for 2 min, 95°C for 10 min, 40 cycles of 95°C for 15 sec, and 60°C for 1 min. All reactions were run in duplicate to reduce confounding variance. U6 snRNA (Applied Biosystems) was used as an internal control. Means from different conditions were compared using the Student’s t-test. A significance threshold of P < 0.05 was used.

### Accession numbers

The sequence data from this study have been submitted to the submitted to the NCBI Gene Expression Omnibus (GEO) ( http://www.ncbi.nlm.nih.gov/geo/) under accession no. GSE32493 All novel microRNA sequences have been submitted to miRBase ( http://www.mirbase.org).

## Competing interests

The authors declare that they have no competing interests.

## Authors’ contributions

SG, HJJ, and FD designed and performed the experiments and SG wrote the manuscript. BC, XT, JP, JHJH, JC, and JV analyzed the data. GA and NB contributed reagents and materials. All authors have read and approved the manuscript for publication. YS conceived and designed the experiments, analyzed the data and wrote the manuscript.

## Supplementary Material

Additional file 1 **Table S1.** Summary of read/sequence counts for miRNA analysis from lymphoblastoid cell lines from centenarians (n = 3) and controls (n = 3). Click here for file

Additional file 2 **Table S2.** Complete list of known (miRBase v 9) average miRNA read counts from lymphoblastoid cells from centenarians (n = 3) and controls (n = 3). All reads present in 50% or more samples with a read number greater than 10. Click here for file

Additional file 3 **Table S3.** Sequencing identified isomiRs from B-Cells of Centenarians and Controls. Click here for file

Additional file 4 **Table S4.** Putative and Confirmed Targets of differentially expressed miRNAs. Click here for file

Additional file 5 **Table S5.** Significantly over-represented GO categories of predicted targets of miRNA differentially expressed in B-Cells of Centenarians as compared to Controls. Click here for file
