# Supplementary material for: Comprehensive microRNA profiling in B-cells of human centenarians by massively parallel sequencing
Source: BMC Genomics. 2012 Jul 31;13:353. doi: 10.1186/1471-2164-13-353 (PMC3563618; doi:10.1186/1471-2164-13-353)
Supplement: Additional file 1 — Table S1. Summary of read/sequence counts for miRNA analysis from lymphoblastoid cell lines from centenarians (n = 3) and controls (n = 3). [file 1471-2164-13-353-S1.pdf]

Supplementary Table S1. Summary of read/sequence counts for miRNA analysis from lymphoblastoid cell lines from centenarians (n=3) and controls (n=3)

| | CONTROLS | | | CENTENARIANS | | |
|---|---|---|---|---|---|---|
| Samples | LG879 | LG1654 | LG3632 | LG1041 | LG4797 | LG6503 |
| Total Reads | 5,001,231 | 5,507,871 | 3,397,339 | 3,416,386 | 5,221,462 | 4,390,079 |
| Poor Quality | -64,961 | -55,831 | -84,198 | -52,886 | -72,897 | -57,902 |
| Improper Size | -976,455 | -1,016,172 | -1,130,632 | -1,420,603 | -1,288,747 | -1,150,913 |
| Reads Meeting Quality and Size | 3,959,815 | 4,435,868 | 2,182,509 | 1,942,897 | 3,859,818 | 3,181,264 |
| Unique Sequences | 276,916 | 254,709 | 151,861 | 184,573 | 226,592 | 235,747 |
| Unique Sequences in Clusters > 1 | 48,199 | 46,301 | 30,659 | 34,832 | 42,318 | 41,654 |
| miRNA/miRNA* identified | 221/55 | | | | | |
| Expanded Total miRNA abundance | 2,559,586 | 3,023,318 | 1,327,913 | 1,033,023 | 2,553,821 | 1,990,772 |
| Ratio of miRNA to Total Reads | 51.18% | 54.89% | 39.09% | 30.24% | 48.91% | 45.35% |
