# Supplementary material for: Comprehensive microRNA profiling in B-cells of human centenarians by massively parallel sequencing
Source: BMC Genomics. 2012 Jul 31;13:353. doi: 10.1186/1471-2164-13-353 (PMC3563618; doi:10.1186/1471-2164-13-353)
Supplement: Additional file 2 — Table S2. Complete list of known (miRBase v 9) average miRNA read counts from lymphoblastoid cells from centenarians (n = 3) and controls (n = 3). All reads present in 50% or more samples with a read number greater than 10. [file 1471-2164-13-353-S2.pdf]

Supplementary Table S2. Complete list of known (miRBase v 9) average miRNA read counts from lymphoblastoid cells from centenarians (n=3) and controls (n=3). All reads present in 50% or more samples with a read number greater than 10.

miRNA	Control	Centenarian	Fold Change(Centenarian/Control)
hsa-let-7a	356790	291190	0.816
hsa-let-7a*	36	32	0.898
hsa-let-7b	21231	15935	0.751
hsa-let-7b*	393	417	1.062
hsa-let-7c	356187	290682	0.816
hsa-let-7d	67492	46456	0.688
hsa-let-7d*	50	43	0.853
hsa-let-7e	2221	1430	0.644
hsa-let-7f	1023496	868104	0.848
hsa-let-7f-1*	16	12	0.771
hsa-let-7g	85634	92025	1.075
hsa-let-7i	33043	37673	1.140
hsa-miR-1	66	53	0.803
hsa-miR-101	5720	5899	1.031
hsa-miR-103	195210	179652	0.920
hsa-miR-103-as	195210	179652	0.920
hsa-miR-106a	1782	2391	1.342
hsa-miR-106b	759	725	0.955
hsa-miR-106b*	214	209	0.974
hsa-miR-107	195084	179541	0.920
hsa-miR-10a	987	1630	1.652
hsa-miR-122	6	34	5.368
hsa-miR-1255a	171	227	1.326
hsa-miR-1255b	171	227	1.326
hsa-miR-1259	35	70	1.981
hsa-miR-1270	71	50	0.708
hsa-miR-1271	23	30	1.319
hsa-miR-1277	16	22	1.327
hsa-miR-1278	44	51	1.150
hsa-miR-128	2056	1720	0.836
hsa-miR-1301	54	55	1.012
hsa-miR-1304	33	23	0.707
hsa-miR-1307	1720	1925	1.119
hsa-miR-130a	107	129	1.199
hsa-miR-130b	1089	932	0.856
hsa-miR-130b*	36	40	1.110

**hsa-miR-132**	47	34	0.723
**hsa-miR-132***	38	29	0.763
**hsa-miR-138**	309	236	0.765
**hsa-miR-140-3p**	50679	49313	0.973
**hsa-miR-142-3p**	1010	1314	1.300
**hsa-miR-142-5p**	26831	24930	0.929
**hsa-miR-146a**	28517	21389	0.750
**hsa-miR-146b-3p**	18	16	0.873
**hsa-miR-146b-5p**	28223	21324	0.756
**hsa-miR-148a**	3866	8528	2.206
**hsa-miR-148a***	113	219	1.935
**hsa-miR-148b**	378	469	1.241
**hsa-miR-148b***	37	46	1.243
**hsa-miR-150**	28	18	0.635
**hsa-miR-150***	53	36	0.681
**hsa-miR-151-3p**	1505	451	0.299
**hsa-miR-151-5p**	86	27	0.311
**hsa-miR-152**	510	565	1.108
**hsa-miR-155**	70851	109615	1.547
**hsa-miR-155***	687	826	1.203
**hsa-miR-15a**	241	231	0.956
**hsa-miR-15b**	814	1017	1.249
**hsa-miR-15b***	176	248	1.411
**hsa-miR-16**	3207	3277	1.022
**hsa-miR-16-2***	283	381	1.344
**hsa-miR-17**	1782	2391	1.342
**hsa-miR-17***	1420	1086	0.765
**hsa-miR-181a**	11197	19438	1.736
**hsa-miR-181a***	209	547	2.617
**hsa-miR-181a-2***	297	449	1.515
**hsa-miR-181b**	8194	13044	1.592
**hsa-miR-181c**	51	103	2.026
**hsa-miR-181d**	889	1321	1.486
**hsa-miR-185**	6798	5605	0.825
**hsa-miR-185***	25	25	1.027
**hsa-miR-186**	1178	1447	1.228
**hsa-miR-18a**	58	70	1.201
**hsa-miR-18a***	12	18	1.543
**hsa-miR-18b**	58	70	1.201
**hsa-miR-191**	35275	24926	0.707

**hsa-miR-192**	2375	4585	1.930
**hsa-miR-193b**	736	1104	1.500
**hsa-miR-193b***	2310	2236	0.968
**hsa-miR-197**	30	36	1.187
**hsa-miR-1974**	1505	4726	3.140
**hsa-miR-1975**	551	1176	2.134
**hsa-miR-1977**	26	42	1.595
**hsa-miR-1979**	1252	2301	1.837
**hsa-miR-199a-3p**	69	77	1.115
**hsa-miR-199b-3p**	69	77	1.115
**hsa-miR-19a**	10	11	1.138
**hsa-miR-19b**	288	323	1.122
**hsa-miR-200c**	89	106	1.195
**hsa-miR-20a**	645	1028	1.595
**hsa-miR-20b**	102	369	3.621
**hsa-miR-20b***	106	236	2.219
**hsa-miR-21**	81191	112017	1.380
**hsa-miR-21***	843	2104	2.496
**hsa-miR-210**	3702	3648	0.986
**hsa-miR-2110**	16	10	0.592
**hsa-miR-215**	2375	4585	1.930
**hsa-miR-219-1-3p**	8	8	1.042
**hsa-miR-22**	538	737	1.371
**hsa-miR-22***	55	63	1.159
**hsa-miR-221**	13870	21440	1.546
**hsa-miR-221***	2006	2218	1.106
**hsa-miR-222**	5227	6609	1.265
**hsa-miR-223**	90	149	1.658
**hsa-miR-223***	193	600	3.103
**hsa-miR-23a**	4925	4055	0.823
**hsa-miR-23a***	38	23	0.600
**hsa-miR-23b**	4921	4055	0.824
**hsa-miR-23b***	59	53	0.888
**hsa-miR-24**	1090	777	0.712
**hsa-miR-25**	37897	46760	1.234
**hsa-miR-25***	3303	3672	1.112
**hsa-miR-26a**	3011	2625	0.872
**hsa-miR-26b**	3220	3310	1.028
**hsa-miR-27a**	1033	1020	0.987
**hsa-miR-27a***	26	36	1.354

**hsa-miR-27b**	1033	1020	0.987
**hsa-miR-27b***	36	57	1.578
**hsa-miR-296-3p**	111	60	0.539
**hsa-miR-29a**	25318	25165	0.994
**hsa-miR-29a***	12	11	0.892
**hsa-miR-29b**	2015	2382	1.182
**hsa-miR-29b-1***	29	42	1.437
**hsa-miR-29c**	2015	2382	1.182
**hsa-miR-29c***	8	9	1.080
**hsa-miR-30a***	4240	5583	1.317
**hsa-miR-30b**	444	734	1.652
**hsa-miR-30b***	99	141	1.432
**hsa-miR-30c**	444	734	1.652
**hsa-miR-30c-1***	67	65	0.980
**hsa-miR-30d**	4039	5024	1.244
**hsa-miR-30e**	1873	2150	1.148
**hsa-miR-30e***	4262	5621	1.319
**hsa-miR-32**	11	11	0.970
**hsa-miR-32***	11	20	1.875
**hsa-miR-320a**	66057	60988	0.923
**hsa-miR-320b**	66033	60962	0.923
**hsa-miR-320c**	66017	60936	0.923
**hsa-miR-324-5p**	36	33	0.925
**hsa-miR-330-3p**	3394	2691	0.793
**hsa-miR-331-3p**	1130	1289	1.141
**hsa-miR-339-3p**	406	428	1.054
**hsa-miR-339-5p**	116	106	0.917
**hsa-miR-33a**	1524	1262	0.828
**hsa-miR-33a***	37	40	1.063
**hsa-miR-33b**	15	15	1.000
**hsa-miR-340**	125	114	0.914
**hsa-miR-342-3p**	1293	1018	0.787
**hsa-miR-342-5p**	131	199	1.522
**hsa-miR-345**	20	76	3.847
**hsa-miR-34a**	85	131	1.545
**hsa-miR-34a***	28	24	0.847
**hsa-miR-34c-5p**	24	21	0.849
**hsa-miR-361-3p**	96	112	1.167
**hsa-miR-361-5p**	102	196	1.912
**hsa-miR-362-3p**	19	13	0.696

**hsa-miR-363**	1577	3990	2.530
**hsa-miR-363***	61	279	4.549
**hsa-miR-365**	689	1000	1.453
**hsa-miR-374a**	106	154	1.461
**hsa-miR-374a***	511	484	0.947
**hsa-miR-374b**	598	591	0.989
**hsa-miR-374b***	598	591	0.989
**hsa-miR-375**	18	20	1.113
**hsa-miR-378**	34251	32777	0.957
**hsa-miR-421**	91	67	0.743
**hsa-miR-423-3p**	1848	2354	1.274
**hsa-miR-423-5p**	22293	21119	0.947
**hsa-miR-424**	96	52	0.540
**hsa-miR-424***	140	90	0.644
**hsa-miR-425**	795	825	1.038
**hsa-miR-425***	103	75	0.727
**hsa-miR-454**	13	42	3.231
**hsa-miR-484**	38	44	1.157
**hsa-miR-486-3p**	241	344	1.425
**hsa-miR-486-5p**	241	344	1.425
**hsa-miR-500***	64	129	2.026
**hsa-miR-501-3p**	20	25	1.230
**hsa-miR-502-3p**	64	129	2.026
**hsa-miR-503**	107	76	0.709
**hsa-miR-505***	124	110	0.892
**hsa-miR-532-3p**	37	36	0.991
**hsa-miR-532-5p**	461	411	0.892
**hsa-miR-542-3p**	18	11	0.642
**hsa-miR-548a-3p**	26	29	1.103
**hsa-miR-548b-5p**	23	25	1.086
**hsa-miR-548d-5p**	23	25	1.086
**hsa-miR-548e**	246	251	1.019
**hsa-miR-548j**	22	24	1.092
**hsa-miR-548k**	48	47	0.986
**hsa-miR-551b**	48	70	1.451
**hsa-miR-551b***	20	34	1.729
**hsa-miR-574-3p**	15	21	1.455
**hsa-miR-576-3p**	35	37	1.057
**hsa-miR-576-5p**	73	116	1.596
**hsa-miR-589**	21	18	0.859

**hsa-miR-590-5p**	26	21	0.818
**hsa-miR-598**	163	183	1.123
**hsa-miR-625**	810	578	0.714
**hsa-miR-625***	810	578	0.714
**hsa-miR-629**	66	105	1.596
**hsa-miR-641**	63	67	1.064
**hsa-miR-642**	133	173	1.295
**hsa-miR-651**	22	30	1.343
**hsa-miR-652**	67	61	0.901
**hsa-miR-660**	47	70	1.482
**hsa-miR-664**	31	41	1.337
**hsa-miR-664***	274	272	0.990
**hsa-miR-7**	490	659	1.345
**hsa-miR-7-1***	64	85	1.328
**hsa-miR-744**	600	533	0.887
**hsa-miR-766**	163	128	0.787
**hsa-miR-769-5p**	14	23	1.659
**hsa-miR-874**	26	29	1.128
**hsa-miR-877**	67	51	0.760
**hsa-miR-886-3p**	623	763	1.225
**hsa-miR-886-5p**	2668	3453	1.294
**hsa-miR-9**	114	210	1.845
**hsa-miR-9***	35	61	1.769
**hsa-miR-92a**	11999	14291	1.191
**hsa-miR-92a-1***	1853	1616	0.872
**hsa-miR-92a-2***	37	41	1.099
**hsa-miR-92b**	145	126	0.869
**hsa-miR-92b***	33	82	2.475
**hsa-miR-93**	2685	3045	1.134
**hsa-miR-93***	7	15	2.190
**hsa-miR-941**	163	238	1.455
**hsa-miR-944**	77	87	1.125
**hsa-miR-98**	1092	1229	1.125
**hsa-miR-99a**	118	59	0.499
**hsa-miR-99b**	32	92	2.845
