# Supplementary material for: Comprehensive microRNA profiling in B-cells of human centenarians by massively parallel sequencing
Source: BMC Genomics. 2012 Jul 31;13:353. doi: 10.1186/1471-2164-13-353 (PMC3563618; doi:10.1186/1471-2164-13-353)
Supplement: Additional file 3 — Table S3. Sequencing identified isomiRs from B-Cells of Centenarians and Controls. [file 1471-2164-13-353-S3.pdf]

Supplementary Table S3: Sequencing identified isomiRs from B-Cells of Centenarians and Controls

miRNA	Sequence
hsa-let-7a	TGAGGTAGTAGGTTGTATAGTT
hsa-let-7a	TGAGGTAGTAGGTTGTATAGTTA
hsa-let-7a	TGAGGTAGTAGGTTGTATAGT
hsa-let-7a	TGAGGTAGTAGGTTGTATAGTTT
hsa-let-7a	TGAGGTAGTAGGTTGTATAGTA
hsa-let-7a	TGAGGTAGTAGGTTGTATAGTTG
hsa-let-7a	TGAGGTAGTAGGTTGTATAGTAA
hsa-let-7a	TGAGGTAGTAGGTTGTATAGTG
hsa-let-7a	TGAGGTAGTAGGTTGTATAGTC
hsa-let-7a	TGAGGTAGTAGGTTGTATAGTTC
hsa-let-7a	TGAGGTAGTAGGTTGTATAGTAT
hsa-let-7a	TGAGGTAGTAGGTTGTATAGTGA
hsa-let-7a	TGAGGTAGTAGGTTGTATAGTCA
hsa-let-7a	TGAGGTAGTAGGTTGTATAGTGT
hsa-let-7a	TGAGGTAGTAGGTTGTATAGTAG
hsa-let-7a	TGAGGTAGTAGGTTGTATAGTCG
hsa-let-7a	TGAGGTAGTAGGTTGTATAGTCT
hsa-let-7a*	CTATACAATCTACTGTCTTTCT
hsa-let-7a*	CTATACAATCTACTGTCTTT
hsa-let-7a*	CTATACAATCTACTGTCTTTTT
hsa-let-7a*	CTATACAATCTACTGTCTTTC
hsa-let-7a*	CTATACAATCTACTGTCTTTT
hsa-let-7a*	CTATACAATCTACTGTCTTTCA
hsa-let-7b	TGAGGTAGTAGGTTGTGTGGTT
hsa-let-7b	TGAGGTAGTAGGTTGTGTGGTTA
hsa-let-7b	TGAGGTAGTAGGTTGTGTGGTTT
hsa-let-7b	TGAGGTAGTAGGTTGTGTGGT
hsa-let-7b	TGAGGTAGTAGGTTGTGTGGTAA
hsa-let-7b	TGAGGTAGTAGGTTGTGTGGTA
hsa-let-7b	TGAGGTAGTAGGTTGTGTGGTAT
hsa-let-7b	TGAGGTAGTAGGTTGTGTGGTTG
hsa-let-7b	TGAGGTAGTAGGTTGTGTGGTAG
hsa-let-7b	TGAGGTAGTAGGTTGTGTGGTG
hsa-let-7b	TGAGGTAGTAGGTTGTGTGGTTC
hsa-let-7b	TGAGGTAGTAGGTTGTGTGGTGA
hsa-let-7b	TGAGGTAGTAGGTTGTGTGGTC
hsa-let-7b	TGAGGTAGTAGGTTGTGTGGTGT
hsa-let-7b	TGAGGTAGTAGGTTGTGTGGTCA
hsa-let-7c	TGAGGTAGTAGGTTGTATGGTT
hsa-let-7c	TGAGGTAGTAGGTTGTATGGTTA
hsa-let-7c	TGAGGTAGTAGGTTGTATGGT
hsa-let-7c	TGAGGTAGTAGGTTGTATGGTTT
hsa-let-7c	TGAGGTAGTAGGTTGTATGGTA

hsa-let-7c	TGAGGTAGTAGGTTGTATGGTAA
hsa-let-7c	TGAGGTAGTAGGTTGTATGGTTG
hsa-let-7c	TGAGGTAGTAGGTTGTATGGTAT
hsa-let-7c	TGAGGTAGTAGGTTGTATGGTC
hsa-let-7d	AGAGGTAGTAGGTTGCATAGTT
hsa-let-7d	AGAGGTAGTAGGTTGCATAGTTA
hsa-let-7d	AGAGGTAGTAGGTTGCATAGT
hsa-let-7d	AGAGGTAGTAGGTTGCATAGTTT
hsa-let-7d	AGAGGTAGTAGGTTGCATAGTA
hsa-let-7d	AGAGGTAGTAGGTTGCATAGTTG
hsa-let-7d	AGAGGTAGTAGGTTGCATAGTAA
hsa-let-7d	AGAGGTAGTAGGTTGCATAGTG
hsa-let-7d	AGAGGTAGTAGGTTGCATAGTC
hsa-let-7d	AGAGGTAGTAGGTTGCATAGTAT
hsa-let-7d	AGAGGTAGTAGGTTGCATAGTTC
hsa-let-7d	AGAGGTAGTAGGTTGCATAGTGA
hsa-let-7d	AGAGGTAGTAGGTTGCATAGTAG
hsa-let-7d	AGAGGTAGTAGGTTGCATAGTCA
hsa-let-7d	AGAGGTAGTAGGTTGCATAGTGT
hsa-let-7d*	CTATACGACCTGCTGCCTTTCT
hsa-let-7d*	CTATACGACCTGCTGCCTTTC
hsa-let-7d*	CTATACGACCTGCTGCCTTTCA
hsa-let-7e	TGAGGTAGGAGGTTGTATAGTT
hsa-let-7e	TGAGGTAGGAGGTTGTATAGTTA
hsa-let-7e	TGAGGTAGGAGGTTGTATAGT
hsa-let-7e	TGAGGTAGGAGGTTGTATAGTTT
hsa-let-7e	TGAGGTAGGAGGTTGTATAGTTG
hsa-let-7e	TGAGGTAGGAGGTTGTATAGTG
hsa-let-7e	TGAGGTAGGAGGTTGTATAGTA
hsa-let-7e	TGAGGTAGGAGGTTGTATAGTAA
hsa-let-7e	TGAGGTAGGAGGTTGTATAGTC
hsa-let-7e	TGAGGTAGGAGGTTGTATAGTGA
hsa-let-7f	TGAGGTAGTAGATTGTATAGTT
hsa-let-7f	TGAGGTAGTAGATTGTATAGTTA
hsa-let-7f	TGAGGTAGTAGATTGTATAGT
hsa-let-7f	TGAGGTAGTAGATTGTATAGTTT
hsa-let-7f	TGAGGTAGTAGATTGTATAGTA
hsa-let-7f	TGAGGTAGTAGATTGTATAGTTG
hsa-let-7f	TGAGGTAGTAGATTGTATAGTAA
hsa-let-7f	TGAGGTAGTAGATTGTATAGTG
hsa-let-7f	TGAGGTAGTAGATTGTATAGTC
hsa-let-7f	TGAGGTAGTAGATTGTATAGTTC
hsa-let-7f	TGAGGTAGTAGATTGTATAGTCA
hsa-let-7f	TGAGGTAGTAGATTGTATAGTAT
hsa-let-7f	TGAGGTAGTAGATTGTATAGTGA
hsa-let-7f	TGAGGTAGTAGATTGTATAGTGT
hsa-let-7f	TGAGGTAGTAGATTGTATAGTAG

hsa-let-7f	TGAGGTAGTAGATTGTATAGTCT
hsa-let-7f	TGAGGTAGTAGATTGTATAGTCG
hsa-let-7f-1*	CTATACAATCTATTGCCTTCCT
hsa-let-7f-1*	CTATACAATCTATTGCCTTCCA
hsa-let-7f-1*	CTATACAATCTATTGCCTTCC
hsa-let-7g	TGAGGTAGTAGTTTGTACAGTT
hsa-let-7g	TGAGGTAGTAGTTTGTACAGTTA
hsa-let-7g	TGAGGTAGTAGTTTGTACAGT
hsa-let-7g	TGAGGTAGTAGTTTGTACAGTTT
hsa-let-7g	TGAGGTAGTAGTTTGTACAGTA
hsa-let-7g	TGAGGTAGTAGTTTGTACAGTTG
hsa-let-7g	TGAGGTAGTAGTTTGTACAGTAA
hsa-let-7g	TGAGGTAGTAGTTTGTACAGTG
hsa-let-7g	TGAGGTAGTAGTTTGTACAGTC
hsa-let-7g	TGAGGTAGTAGTTTGTACAGTTC
hsa-let-7g	TGAGGTAGTAGTTTGTACAGTAT
hsa-let-7g	TGAGGTAGTAGTTTGTACAGTGA
hsa-let-7g	TGAGGTAGTAGTTTGTACAGTCA
hsa-let-7g*	CTGTACAGGCCACTGCCTTGCT
hsa-let-7i	TGAGGTAGTAGTTTGTGCTGTT
hsa-let-7i	TGAGGTAGTAGTTTGTGCTGTTA
hsa-let-7i	TGAGGTAGTAGTTTGTGCTGT
hsa-let-7i	TGAGGTAGTAGTTTGTGCTGTTT
hsa-let-7i	TGAGGTAGTAGTTTGTGCTGTAA
hsa-let-7i	TGAGGTAGTAGTTTGTGCTGTA
hsa-let-7i	TGAGGTAGTAGTTTGTGCTGTTG
hsa-let-7i	TGAGGTAGTAGTTTGTGCTGTG
hsa-let-7i	TGAGGTAGTAGTTTGTGCTGTAT
hsa-let-7i	TGAGGTAGTAGTTTGTGCTGTC
hsa-let-7i	TGAGGTAGTAGTTTGTGCTGTAG
hsa-let-7i	TGAGGTAGTAGTTTGTGCTGTTC
hsa-let-7i	TGAGGTAGTAGTTTGTGCTGTGA
hsa-let-7i	TGAGGTAGTAGTTTGTGCTGTGT
hsa-let-7i*	CTGCGCAAGCTACTGCCTTGCT
hsa-miR-1	TGGAATGTAAAGAAGTATGTAT
hsa-miR-1	TGGAATGTAAAGAAGTATGTA
hsa-miR-101	TACAGTACTGTGATAACTGAA
hsa-miR-101	TACAGTACTGTGATAACTGAAG
hsa-miR-101	TACAGTACTGTGATAACTGAAA
hsa-miR-101	TACAGTACTGTGATAACTGA
hsa-miR-101	TACAGTACTGTGATAACTGAAGA
hsa-miR-101	TACAGTACTGTGATAACTGAAT
hsa-miR-101	TACAGTACTGTGATAACTGAAAA
hsa-miR-101	TACAGTACTGTGATAACTGAAGT
hsa-miR-101	TACAGTACTGTGATAACTGAT
hsa-miR-101	TACAGTACTGTGATAACTGATT
hsa-miR-101	TACAGTACTGTGATAACTGAAC

hsa-miR-103	AGCAGCATTGTACAGGGCTATGA
hsa-miR-103	AGCAGCATTGTACAGGGCTATG
hsa-miR-103	AGCAGCATTGTACAGGGCTATGT
hsa-miR-103	AGCAGCATTGTACAGGGCTATGG
hsa-miR-103	AGCAGCATTGTACAGGGCTATGC
hsa-miR-103-2*	AGCTTCTTTACAGTGCTGCCTTG
hsa-miR-106a	AAAAGTGCTTACAGTGCAGGTAG
hsa-miR-106a	AAAAGTGCTTACAGTGCAGGTA
hsa-miR-106a	AAAAGTGCTTACAGTGCAGGTAA
hsa-miR-106a	AAAAGTGCTTACAGTGCAGGTAT
hsa-miR-106b	TAAAGTGCTGACAGTGCAGATA
hsa-miR-106b	TAAAGTGCTGACAGTGCAGAT
hsa-miR-106b	TAAAGTGCTGACAGTGCAGATAA
hsa-miR-106b	TAAAGTGCTGACAGTGCAGA
hsa-miR-106b	TAAAGTGCTGACAGTGCAGATAT
hsa-miR-106b	TAAAGTGCTGACAGTGCAGATAG
hsa-miR-106b	TAAAGTGCTGACAGTGCAGATT
hsa-miR-106b	TAAAGTGCTGACAGTGCAGAA
hsa-miR-106b	TAAAGTGCTGACAGTGCAGAGA
hsa-miR-106b	TAAAGTGCTGACAGTGCAGAAA
hsa-miR-106b*	CCGCACTGTGGGTACTTGCTG
hsa-miR-106b*	CCGCACTGTGGGTACTTGCTGA
hsa-miR-106b*	CCGCACTGTGGGTACTTGCTGAA
hsa-miR-106b*	CCGCACTGTGGGTACTTGCTGC
hsa-miR-106b*	CCGCACTGTGGGTACTTGCTGT
hsa-miR-107	AGCAGCATTGTACAGGGCTATC
hsa-miR-107	AGCAGCATTGTACAGGGCTATCA
hsa-miR-107	AGCAGCATTGTACAGGGCTATCT
hsa-miR-107	AGCAGCATTGTACAGGGCTATCG
hsa-miR-10a	TACCCTGTAGATCCGAATTTGT
hsa-miR-10a	TACCCTGTAGATCCGAATTTGTG
hsa-miR-10a	TACCCTGTAGATCCGAATTTGTA
hsa-miR-10a	TACCCTGTAGATCCGAATTTGTT
hsa-miR-10a*	CAAATTCGTATCTAGGGGAAT
hsa-miR-122	TGGAGTGTGACAATGGTGTTT
hsa-miR-1246	AATGGATTTTTGGAGCAGGGA
hsa-miR-1246	AATGGATTTTTGGAGCAGGG
hsa-miR-1246	AATGGATTTTTGGAGCAGGGAG
hsa-miR-1246	AATGGATTTTTGGAGCAGGGAGA
hsa-miR-1246	AATGGATTTTTGGAGCAGG
hsa-miR-1246	AATGGATTTTTGGAGCAGGGAA
hsa-miR-1246	AATGGATTTTTGGAGCAG
hsa-miR-1246	AATGGATTTTTGGAGCAGGGAAA
hsa-miR-1246	AATGGATTTTTGGAGCAGGAA
hsa-miR-1249	ACGCCCTTCCCCCCCTTCTTCA
hsa-miR-1254	AGCCTGGAAGCTGGAGCCTGCAG
hsa-miR-1255a	AGGATGAGCAAAGAAAGTAGATT

hsa-miR-1255a	AGGATGAGCAAAGAAAGTAGAT
hsa-miR-1255a	AGGATGAGCAAAGAAAGTAGATA
hsa-miR-1255b	CGGATGAGCAAAGAAAGTGGTT
hsa-miR-1255b	CGGATGAGCAAAGAAAGTGGTTT
hsa-miR-1255b	CGGATGAGCAAAGAAAGTGGTTA
hsa-miR-1255b	CGGATGAGCAAAGAAAGTGGT
hsa-miR-1256	AGGCATTGACTTCTCACTAGCT
hsa-miR-125a-5p	TCCCTGAGACCCTTTAACCTGTG
hsa-miR-125b	TCCCTGAGACCCTAACTTGTGA
hsa-miR-125b	TCCCTGAGACCCTAACTTGTG
hsa-miR-1260	ATCCCACCTCTGCCACCA
hsa-miR-1260b	ATCCCACCACTGCCACCA
hsa-miR-1268	CGGGCGTGGTGGTGGGGG
hsa-miR-1268	CGGGCGTGGTGGTGGGGGTGGG
hsa-miR-1268	CGGGCGTGGTGGTGGGG
hsa-miR-1268	CGGGCGTGGTGGTGGGGGA
hsa-miR-1268	CGGGCGTGGTGGTGGGGGAA
hsa-miR-1270	CTGGAGATATGGAAGAGCTGTGT
hsa-miR-1270	CTGGAGATATGGAAGAGCTGTG
hsa-miR-1270	CTGGAGATATGGAAGAGCTGTGA
hsa-miR-1271	CTTGGCACCTAGCAAGCACTCA
hsa-miR-1271	CTTGGCACCTAGCAAGCACTC
hsa-miR-1273c	GGCGACAAAACGAGACCCTGT
hsa-miR-1273c	GGCGACAAAACGAGACCCTGTCA
hsa-miR-1274a	GTCCCTGTTCAGGCGCCA
hsa-miR-1274b	TCCCTGTTCGGGCGCCA
hsa-miR-1274b	TCCCTGTTCGGGCGCC
hsa-miR-1274b	TCCCTGTTCGGGCGCCC
hsa-miR-1275	GTGGGGGAGAGGCTGTA
hsa-miR-1275	GTGGGGGAGAGGCTGT
hsa-miR-1275	GTGGGGGAGAGGCTGTC
hsa-miR-1275	GTGGGGGAGAGGCTGTAA
hsa-miR-1275	GTGGGGGAGAGGCTGTAAA
hsa-miR-1275	GTGGGGGAGAGGCTGTCGA
hsa-miR-1275	GTGGGGGAGAGGCTGTCA
hsa-miR-1275	GTGGGGGAGAGGCTGTCG
hsa-miR-1275	GTGGGGGAGAGGCTGTCGCT
hsa-miR-1275	GTGGGGGAGAGGCTGTCAA
hsa-miR-1275	GTGGGGGAGAGGCTGTAAAA
hsa-miR-1275	GTGGGGGAGAGGCTGTCGC
hsa-miR-1275	GTGGGGGAGAGGCTGTCGCTG
hsa-miR-1275	GTGGGGGAGAGGCTGTT
hsa-miR-1275	GTGGGGGAGAGGCTGTCGAA
hsa-miR-1275	GTGGGGGAGAGGCTGTAAAAA
hsa-miR-1275	GTGGGGGAGAGGCTGTCGCA
hsa-miR-1275	GTGGGGGAGAGGCTGTG
hsa-miR-1275	GTGGGGGAGAGGCTGTCGCTA

hsa-miR-1275	GTGGGGGAGAGGCTGTCGG
hsa-miR-1275	GTGGGGGAGAGGCTGTGA
hsa-miR-1275	GTGGGGGAGAGGCTGTCGAAA
hsa-miR-1275	GTGGGGGAGAGGCTGTCAAA
hsa-miR-1275	GTGGGGGAGAGGCTGTAT
hsa-miR-1276	TAAAGAGCCCTGTGGAGACA
hsa-miR-1277	TACGTAGATATATATGTATTTT
hsa-miR-1277	TACGTAGATATATATGTATTT
hsa-miR-1278	TAGTACTGTGCATATCATCTAT
hsa-miR-1278	TAGTACTGTGCATATCATCTA
hsa-miR-128	TCACAGTGAACCGGTCTCTTT
hsa-miR-128	TCACAGTGAACCGGTCTCTTTA
hsa-miR-128	TCACAGTGAACCGGTCTCTT
hsa-miR-128	TCACAGTGAACCGGTCTCTTTT
hsa-miR-128	TCACAGTGAACCGGTCTCTTA
hsa-miR-128	TCACAGTGAACCGGTCTCTTTAA
hsa-miR-128	TCACAGTGAACCGGTCTCTTAA
hsa-miR-128	TCACAGTGAACCGGTCTCTTTTA
hsa-miR-128	TCACAGTGAACCGGTCTCTTTTT
hsa-miR-128	TCACAGTGAACCGGTCTCTTTAT
hsa-miR-128	TCACAGTGAACCGGTCTCTTAT
hsa-miR-128	TCACAGTGAACCGGTCTCTTTG
hsa-miR-128	TCACAGTGAACCGGTCTCTTAAA
hsa-miR-128	TCACAGTGAACCGGTCTCTTG
hsa-miR-128	TCACAGTGAACCGGTCTCTTTAG
hsa-miR-128	TCACAGTGAACCGGTCTCTTAG
hsa-miR-1280	TCCCACCGCTGCCACCA
hsa-miR-1285	TCTGGGCAACAAAGTGAGACCT
hsa-miR-1285	TCTGGGCAACAAAGTGAGACC
hsa-miR-1285	TCTGGGCAACAAAGTGAGACCTT
hsa-miR-1285	TCTGGGCAACAAAGTGAGACCTA
hsa-miR-1287	TGCTGGATCAGTGGTTCGAGT
hsa-miR-129-3p	AAGCCCTTACCCCAAAAAGCAT
hsa-miR-1294	TGTGAGGTTGGCATTGTTGTCT
hsa-miR-1294	TGTGAGGTTGGCATTGTTGTC
hsa-miR-129-5p	CTTTTTGCGGTCTGGGCTTGC
hsa-miR-129-5p	CTTTTTGCGGTCTGGGCTTG
hsa-miR-1299	TTCTGGAATTCTGTGTGAGGGA
hsa-miR-1301	TTGCAGCTGCCTGGGAGTGACTT
hsa-miR-1304	TTTGAGGCTACAGTGAGATGTG
hsa-miR-1306	ACGTTGGCTCTGGTGGTGATG
hsa-miR-1306	ACGTTGGCTCTGGTGGTGAT
hsa-miR-1306	ACGTTGGCTCTGGTGGTGATGT
hsa-miR-1307	ACTCGGCGTGGCGTCGGTCGTG
hsa-miR-1307	ACTCGGCGTGGCGTCGGTCGTGG
hsa-miR-1307	ACTCGGCGTGGCGTCGGTCGT
hsa-miR-1307	ACTCGGCGTGGCGTCGGTCGTGT

hsa-miR-1307	ACTCGGCGTGGCGTCGGTCGTGA
hsa-miR-1307	ACTCGGCGTGGCGTCGGTCGTT
hsa-miR-1307	ACTCGGCGTGGCGTCGGTCGTA
hsa-miR-1307	ACTCGGCGTGGCGTCGGTCGTAA
hsa-miR-1307	ACTCGGCGTGGCGTCGGTCGTAT
hsa-miR-130a	CAGTGCAATGTTAAAAGGGCAT
hsa-miR-130a	CAGTGCAATGTTAAAAGGGCAC
hsa-miR-130a	CAGTGCAATGTTAAAAGGGCATT
hsa-miR-130a	CAGTGCAATGTTAAAAGGGCA
hsa-miR-130a	CAGTGCAATGTTAAAAGGGCATA
hsa-miR-130a	CAGTGCAATGTTAAAAGGGCACA
hsa-miR-130a	CAGTGCAATGTTAAAAGGGCACT
hsa-miR-130a	CAGTGCAATGTTAAAAGGGCAA
hsa-miR-130a	CAGTGCAATGTTAAAAGGGCACC
hsa-miR-130b	CAGTGCAATGATGAAAGGGCAT
hsa-miR-130b	CAGTGCAATGATGAAAGGGCA
hsa-miR-130b	CAGTGCAATGATGAAAGGGCAA
hsa-miR-130b	CAGTGCAATGATGAAAGGGCATT
hsa-miR-130b	CAGTGCAATGATGAAAGGGCATA
hsa-miR-130b	CAGTGCAATGATGAAAGGGCAAA
hsa-miR-130b	CAGTGCAATGATGAAAGGGCAG
hsa-miR-130b	CAGTGCAATGATGAAAGGGCAAT
hsa-miR-130b	CAGTGCAATGATGAAAGGGCATG
hsa-miR-130b*	ACTCTTTCCCTGTTGCACTACT
hsa-miR-130b*	ACTCTTTCCCTGTTGCACTA
hsa-miR-130b*	ACTCTTTCCCTGTTGCACTAC
hsa-miR-132	TAACAGTCTACAGCCATGGTCG
hsa-miR-132	TAACAGTCTACAGCCATGGTCGT
hsa-miR-132	TAACAGTCTACAGCCATGGTC
hsa-miR-132	TAACAGTCTACAGCCATGGTCGA
hsa-miR-132*	ACCGTGGCTTTCGATTGTTACT
hsa-miR-138	AGCTGGTGTTGTGAATCAGGCCG
hsa-miR-138	AGCTGGTGTTGTGAATCAGGCC
hsa-miR-140-3p	TACCACAGGGTAGAACCACGGA
hsa-miR-140-3p	TACCACAGGGTAGAACCACGGAC
hsa-miR-140-3p	TACCACAGGGTAGAACCACGGAA
hsa-miR-140-3p	TACCACAGGGTAGAACCACGGAT
hsa-miR-140-3p	TACCACAGGGTAGAACCACGG
hsa-miR-140-3p	TACCACAGGGTAGAACCACGGAG
hsa-miR-140-3p	TACCACAGGGTAGAACCACG
hsa-miR-140-3p	TACCACAGGGTAGAACCACGGT
hsa-miR-140-3p	TACCACAGGGTAGAACCACGTA
hsa-miR-140-3p	TACCACAGGGTAGAACCACGGTA
hsa-miR-140-3p	TACCACAGGGTAGAACCACGGCC
hsa-miR-140-3p	TACCACAGGGTAGAACCACGGTT
hsa-miR-140-3p	TACCACAGGGTAGAACCACGTAC
hsa-miR-140-3p	TACCACAGGGTAGAACCACGGC

hsa-miR-140-3p	TACCACAGGGTAGAACCACGGCA
hsa-miR-140-3p	TACCACAGGGTAGAACCACGTAA
hsa-miR-140-3p	TACCACAGGGTAGAACCACGGG
hsa-miR-140-3p	TACCACAGGGTAGAACCACGA
hsa-miR-140-3p	TACCACAGGGTAGAACCACGT
hsa-miR-140-3p	TACCACAGGGTAGAACCACGAAA
hsa-miR-140-3p	TACCACAGGGTAGAACCACGGTC
hsa-miR-140-5p	CAGTGGTTTTACCCTATGGTAG
hsa-miR-140-5p	CAGTGGTTTTACCCTATGGTA
hsa-miR-140-5p	CAGTGGTTTTACCCTATGGTAA
hsa-miR-140-5p	CAGTGGTTTTACCCTATGGTAGA
hsa-miR-142-3p	TGTAGTGTTTCCTACTTTATGG
hsa-miR-142-3p	TGTAGTGTTTCCTACTTTATGGA
hsa-miR-142-3p	TGTAGTGTTTCCTACTTTATGGT
hsa-miR-142-5p	CATAAAGTAGAAAGCACTACT
hsa-miR-142-5p	CATAAAGTAGAAAGCACTACTA
hsa-miR-142-5p	CATAAAGTAGAAAGCACTAC
hsa-miR-142-5p	CATAAAGTAGAAAGCACTACTAA
hsa-miR-142-5p	CATAAAGTAGAAAGCACTACTAT
hsa-miR-142-5p	CATAAAGTAGAAAGCACTACA
hsa-miR-142-5p	CATAAAGTAGAAAGCACTACTAG
hsa-miR-142-5p	CATAAAGTAGAAAGCACTACC
hsa-miR-142-5p	CATAAAGTAGAAAGCACTACG
hsa-miR-142-5p	CATAAAGTAGAAAGCACTACAA
hsa-miR-142-5p	CATAAAGTAGAAAGCACTACGA
hsa-miR-142-5p	CATAAAGTAGAAAGCACTACTT
hsa-miR-143	TGAGATGAAGCACTGTAGCT
hsa-miR-146a	TGAGAACTGAATTCCATGGGTT
hsa-miR-146a	TGAGAACTGAATTCCATGGGTTA
hsa-miR-146a	TGAGAACTGAATTCCATGGGTTG
hsa-miR-146a	TGAGAACTGAATTCCATGGGTTT
hsa-miR-146a	TGAGAACTGAATTCCATGGGT
hsa-miR-146a	TGAGAACTGAATTCCATGGGTAA
hsa-miR-146a	TGAGAACTGAATTCCATGGGTA
hsa-miR-146a	TGAGAACTGAATTCCATGGGTAT
hsa-miR-146a	TGAGAACTGAATTCCATGGGTG
hsa-miR-146a	TGAGAACTGAATTCCATGGGTAG
hsa-miR-146a	TGAGAACTGAATTCCATGGGTC
hsa-miR-146a	TGAGAACTGAATTCCATGGGTTC
hsa-miR-146a	TGAGAACTGAATTCCATGGGTGG
hsa-miR-146a	TGAGAACTGAATTCCATGGGTGA
hsa-miR-146a	TGAGAACTGAATTCCATGGGTCA
hsa-miR-146a	TGAGAACTGAATTCCATGGGTGT
hsa-miR-146b-5p	TGAGAACTGAATTCCATAGGCTG
hsa-miR-146b-5p	TGAGAACTGAATTCCATAGGCT
hsa-miR-146b-5p	TGAGAACTGAATTCCATAGGC
hsa-miR-146b-5p	TGAGAACTGAATTCCATAGGCTA

hsa-miR-146b-5p	TGAGAACTGAATTCCATAGGCA
hsa-miR-146b-5p	TGAGAACTGAATTCCATAGGCTT
hsa-miR-146b-5p	TGAGAACTGAATTCCATAGGCAA
hsa-miR-146b-5p	TGAGAACTGAATTCCATAGGCGG
hsa-miR-146b-5p	TGAGAACTGAATTCCATAGGCG
hsa-miR-148a	TCAGTGCACTACAGAACTTTGT
hsa-miR-148a	TCAGTGCACTACAGAACTTTG
hsa-miR-148a	TCAGTGCACTACAGAACTTTGAA
hsa-miR-148a	TCAGTGCACTACAGAACTTTGA
hsa-miR-148a	TCAGTGCACTACAGAACTTTGTC
hsa-miR-148a	TCAGTGCACTACAGAACTTTGTT
hsa-miR-148a	TCAGTGCACTACAGAACTTTGTA
hsa-miR-148a	TCAGTGCACTACAGAACTTTGG
hsa-miR-148a	TCAGTGCACTACAGAACTTTGC
hsa-miR-148a*	AAAGTTCTGAGACACTCCGACT
hsa-miR-148a*	AAAGTTCTGAGACACTCCGAC
hsa-miR-148b	TCAGTGCATCACAGAACTTTGT
hsa-miR-148b	TCAGTGCATCACAGAACTTTG
hsa-miR-148b	TCAGTGCATCACAGAACTTTGTT
hsa-miR-148b	TCAGTGCATCACAGAACTTTGAA
hsa-miR-148b	TCAGTGCATCACAGAACTTTGA
hsa-miR-148b	TCAGTGCATCACAGAACTTTGTA
hsa-miR-148b*	AAGTTCTGTTATACACTCAGGCT
hsa-miR-150	TCTCCCAACCCTTGTACCAGTG
hsa-miR-150	TCTCCCAACCCTTGTACCAGTGT
hsa-miR-150	TCTCCCAACCCTTGTACCAGT
hsa-miR-150	TCTCCCAACCCTTGTACCAGTGA
hsa-miR-150*	CTGGTACAGGCCTGGGGGACA
hsa-miR-151-3p	CTAGACTGAAGCTCCTTGAGGAA
hsa-miR-151-3p	CTAGACTGAAGCTCCTTGAGGA
hsa-miR-151-3p	CTAGACTGAAGCTCCTTGAGG
hsa-miR-151-3p	CTAGACTGAAGCTCCTTGAGGT
hsa-miR-151-3p	CTAGACTGAAGCTCCTTGAGGAT
hsa-miR-151-3p	CTAGACTGAAGCTCCTTGAG
hsa-miR-151-3p	CTAGACTGAAGCTCCTTGAGGTA
hsa-miR-151-3p	CTAGACTGAAGCTCCTTGAGA
hsa-miR-151-3p	CTAGACTGAAGCTCCTTGAGAA
hsa-miR-151-3p	CTAGACTGAAGCTCCTTGAGGAG
hsa-miR-151-3p	CTAGACTGAAGCTCCTTGAGT
hsa-miR-151-3p	CTAGACTGAAGCTCCTTGAGAAA
hsa-miR-151-3p	CTAGACTGAAGCTCCTTGAGGTT
hsa-miR-151-3p	CTAGACTGAAGCTCCTTGAGGAC
hsa-miR-151-3p	CTAGACTGAAGCTCCTTGAGGC
hsa-miR-151-3p	CTAGACTGAAGCTCCTTGAGGCA
hsa-miR-151-3p	CTAGACTGAAGCTCCTTGAGTAA
hsa-miR-151-5p	TCGAGGAGCTCACAGTCTAGTA
hsa-miR-151-5p	TCGAGGAGCTCACAGTCTAGT

hsa-miR-151-5p	TCGAGGAGCTCACAGTCTAGTAA
hsa-miR-151-5p	TCGAGGAGCTCACAGTCTAG
hsa-miR-151-5p	TCGAGGAGCTCACAGTCTAGTAT
hsa-miR-152	TCAGTGCATGACAGAACTTGG
hsa-miR-152	TCAGTGCATGACAGAACTTGGG
hsa-miR-152	TCAGTGCATGACAGAACTTGGT
hsa-miR-152	TCAGTGCATGACAGAACTTGGGT
hsa-miR-152	TCAGTGCATGACAGAACTTGGGA
hsa-miR-152	TCAGTGCATGACAGAACTTGGA
hsa-miR-152	TCAGTGCATGACAGAACTTGGTT
hsa-miR-152	TCAGTGCATGACAGAACTTGGTA
hsa-miR-152	TCAGTGCATGACAGAACTTG
hsa-miR-152	TCAGTGCATGACAGAACTTGT
hsa-miR-155	TTAATGCTAATCGTGATAGGGGT
hsa-miR-155	TTAATGCTAATCGTGATAGGGG
hsa-miR-155	TTAATGCTAATCGTGATAGGGGA
hsa-miR-155	TTAATGCTAATCGTGATAGGGGG
hsa-miR-155	TTAATGCTAATCGTGATAGGGGC
hsa-miR-155*	CTCCTACATATTAGCATTAACA
hsa-miR-155*	CTCCTACATATTAGCATTAAC
hsa-miR-155*	CTCCTACATATTAGCATTAACT
hsa-miR-155*	CTCCTACATATTAGCATTAACAA
hsa-miR-155*	CTCCTACATATTAGCATTAACC
hsa-miR-15a	TAGCAGCACATAATGGTTTGTG
hsa-miR-15a	TAGCAGCACATAATGGTTTGT
hsa-miR-15a	TAGCAGCACATAATGGTTTGTGA
hsa-miR-15a	TAGCAGCACATAATGGTTTGTGT
hsa-miR-15a	TAGCAGCACATAATGGTTTGTA
hsa-miR-15a	TAGCAGCACATAATGGTTTGTGG
hsa-miR-15b	TAGCAGCACATCATGGTTTACA
hsa-miR-15b	TAGCAGCACATCATGGTTTAC
hsa-miR-15b	TAGCAGCACATCATGGTTTACAA
hsa-miR-15b*	CGAATCATTATTTGCTGCTCTA
hsa-miR-15b*	CGAATCATTATTTGCTGCTCT
hsa-miR-15b*	CGAATCATTATTTGCTGCTCTAA
hsa-miR-15b*	CGAATCATTATTTGCTGCTCTAT
hsa-miR-16	TAGCAGCACGTAAATATTGGCG
hsa-miR-16	TAGCAGCACGTAAATATTGGCGT
hsa-miR-16	TAGCAGCACGTAAATATTGGC
hsa-miR-16	TAGCAGCACGTAAATATTGGCGA
hsa-miR-16	TAGCAGCACGTAAATATTGGCA
hsa-miR-16	TAGCAGCACGTAAATATTGGCAA
hsa-miR-16	TAGCAGCACGTAAATATTGGCT
hsa-miR-16-1*	CCAGTATTAACTGTGCTGCTGA
hsa-miR-16-1*	CCAGTATTAACTGTGCTGCTGAA
hsa-miR-16-2*	CCAATATTACTGTGCTGCTTTA
hsa-miR-16-2*	CCAATATTACTGTGCTGCTTT

hsa-miR-16-2*	CCAATATTACTGTGCTGCTTTAA
hsa-miR-16-2*	CCAATATTACTGTGCTGCTTTT
hsa-miR-17	CAAAGTGCTTACAGTGCAGGTAG
hsa-miR-17	CAAAGTGCTTACAGTGCAGGTA
hsa-miR-17	CAAAGTGCTTACAGTGCAGGTAA
hsa-miR-17	CAAAGTGCTTACAGTGCAGGTAT
hsa-miR-17*	ACTGCAGTGAAGGCACTTGTAG
hsa-miR-17*	ACTGCAGTGAAGGCACTTGTAGA
hsa-miR-17*	ACTGCAGTGAAGGCACTTGTAGC
hsa-miR-17*	ACTGCAGTGAAGGCACTTGTA
hsa-miR-17*	ACTGCAGTGAAGGCACTTGTAGT
hsa-miR-17*	ACTGCAGTGAAGGCACTTGTAA
hsa-miR-17*	ACTGCAGTGAAGGCACTTGTAT
hsa-miR-181a	AACATTCAACGCTGTCGGTGAGT
hsa-miR-181a	AACATTCAACGCTGTCGGTGAG
hsa-miR-181a	AACATTCAACGCTGTCGGTGAGA
hsa-miR-181a	AACATTCAACGCTGTCGGTGAGG
hsa-miR-181a	AACATTCAACGCTGTCGGTGAGC
hsa-miR-181a*	ACCATCGACCGTTGATTGTACC
hsa-miR-181a*	ACCATCGACCGTTGATTGTAC
hsa-miR-181a*	ACCATCGACCGTTGATTGTACT
hsa-miR-181a-2*	ACCACTGACCGTTGACTGTAC
hsa-miR-181a-2*	ACCACTGACCGTTGACTGTACC
hsa-miR-181a-2*	ACCACTGACCGTTGACTGTACT
hsa-miR-181b	AACATTCATTGCTGTCGGTGGGT
hsa-miR-181b	AACATTCATTGCTGTCGGTGGG
hsa-miR-181b	AACATTCATTGCTGTCGGTGGGA
hsa-miR-181b	AACATTCATTGCTGTCGGTGGGG
hsa-miR-181b	AACATTCATTGCTGTCGGTGGGC
hsa-miR-181c	AACATTCAACCTGTCGGTGAGTT
hsa-miR-181c	AACATTCAACCTGTCGGTGAGT
hsa-miR-181c	AACATTCAACCTGTCGGTGAG
hsa-miR-181d	AACATTCATTGTTGTCGGTGGGT
hsa-miR-181d	AACATTCATTGTTGTCGGTGGG
hsa-miR-181d	AACATTCATTGTTGTCGGTGGGA
hsa-miR-1827	TGAGGCAGTAGATTGAATAGTT
hsa-miR-1827	TGAGGCAGTAGATTGAATAGGT
hsa-miR-184	TGGACGGAGAACTGATAAGGGC
hsa-miR-185	TGGAGAGAAAGGCAGTTCCTGA
hsa-miR-185	TGGAGAGAAAGGCAGTTCCTGAA
hsa-miR-185	TGGAGAGAAAGGCAGTTCCTGAT
hsa-miR-185	TGGAGAGAAAGGCAGTTCCTG
hsa-miR-185	TGGAGAGAAAGGCAGTTCCTGT
hsa-miR-185	TGGAGAGAAAGGCAGTTCCTGAG
hsa-miR-185	TGGAGAGAAAGGCAGTTCCTGTA
hsa-miR-185	TGGAGAGAAAGGCAGTTCCTGC
hsa-miR-185	TGGAGAGAAAGGCAGTTCCTGAC

hsa-miR-185	TGGAGAGAAAGGCAGTTCCTGG
hsa-miR-185	TGGAGAGAAAGGCAGTTCCTGTT
hsa-miR-185*	AGGGGCTGGCTTTCCTCTGGT
hsa-miR-185*	AGGGGCTGGCTTTCCTCTGGTA
hsa-miR-186	CAAAGAATTCTCCTTTTGGGCT
hsa-miR-186	CAAAGAATTCTCCTTTTGGGCTT
hsa-miR-186	CAAAGAATTCTCCTTTTGGGC
hsa-miR-186	CAAAGAATTCTCCTTTTGGGCTA
hsa-miR-186	CAAAGAATTCTCCTTTTGGGCA
hsa-miR-186	CAAAGAATTCTCCTTTTGGGCAT
hsa-miR-186	CAAAGAATTCTCCTTTTGGGCAA
hsa-miR-186	CAAAGAATTCTCCTTTTGGGCTG
hsa-miR-186	CAAAGAATTCTCCTTTTGGGCG
hsa-miR-188-5p	CATCCCTTGCATGGTGGAGGGAA
hsa-miR-188-5p	CATCCCTTGCATGGTGGAGGGTA
hsa-miR-18a	TAAGGTGCATCTAGTGCAGATAG
hsa-miR-18a	TAAGGTGCATCTAGTGCAGATA
hsa-miR-18a*	ACTGCCCTAAGTGCTCCTTCTG
hsa-miR-18a*	ACTGCCCTAAGTGCTCCTTCTGT
hsa-miR-18a*	ACTGCCCTAAGTGCTCCTTCTGA
hsa-miR-1908	CGGCGGGGACGGCGATTGGTC
hsa-miR-1908	CGGCGGGGACGGCGATTGGT
hsa-miR-191	CAACGGAATCCCAAAAGCAGCTG
hsa-miR-191	CAACGGAATCCCAAAAGCAGCT
hsa-miR-191	CAACGGAATCCCAAAAGCAGCTA
hsa-miR-191	CAACGGAATCCCAAAAGCAGCTT
hsa-miR-191	CAACGGAATCCCAAAAGCAGCTC
hsa-miR-191*	GCTGCGCTTGGATTTCGTCCC
hsa-miR-192	CTGACCTATGAATTGACAGCC
hsa-miR-192	CTGACCTATGAATTGACAGCCA
hsa-miR-192	CTGACCTATGAATTGACAGC
hsa-miR-192	CTGACCTATGAATTGACAGCCAT
hsa-miR-192	CTGACCTATGAATTGACAGCCAA
hsa-miR-192	CTGACCTATGAATTGACAGCCT
hsa-miR-192	CTGACCTATGAATTGACAGCCAG
hsa-miR-192	CTGACCTATGAATTGACAGCA
hsa-miR-192	CTGACCTATGAATTGACAGCT
hsa-miR-192	CTGACCTATGAATTGACAGCCG
hsa-miR-192	CTGACCTATGAATTGACAGCAA
hsa-miR-192	CTGACCTATGAATTGACAGCCC
hsa-miR-192	CTGACCTATGAATTGACAGCG
hsa-miR-193b	AACTGGCCCTCAAAGTCCCGCT
hsa-miR-193b	AACTGGCCCTCAAAGTCCCGCTT
hsa-miR-193b	AACTGGCCCTCAAAGTCCCGC
hsa-miR-193b	AACTGGCCCTCAAAGTCCCGCTA
hsa-miR-193b	AACTGGCCCTCAAAGTCCCGCA
hsa-miR-193b	AACTGGCCCTCAAAGTCCCGCC

hsa-miR-193b	AACTGGCCCTCAAAGTCCCGCCA
hsa-miR-193b	AACTGGCCCTCAAAGTCCCGCAA
hsa-miR-193b	AACTGGCCCTCAAAGTCCCGCAT
hsa-miR-193b	AACTGGCCCTCAAAGTCCCGCCT
hsa-miR-193b	AACTGGCCCTCAAAGTCCCGCTG
hsa-miR-193b	AACTGGCCCTCAAAGTCCCGCG
hsa-miR-193b*	CGGGGTTTTGAGGGCGAGATGA
hsa-miR-193b*	CGGGGTTTTGAGGGCGAGATG
hsa-miR-193b*	CGGGGTTTTGAGGGCGAGATGT
hsa-miR-193b*	CGGGGTTTTGAGGGCGAGATGAA
hsa-miR-193b*	CGGGGTTTTGAGGGCGAGATGAT
hsa-miR-193b*	CGGGGTTTTGAGGGCGAGATGTT
hsa-miR-193b*	CGGGGTTTTGAGGGCGAGATGTA
hsa-miR-193b*	CGGGGTTTTGAGGGCGAGATGAG
hsa-miR-193b*	CGGGGTTTTGAGGGCGAGATGC
hsa-miR-194	TGTAACAGCAACTCCATGTGGAA
hsa-miR-194	TGTAACAGCAACTCCATGTGGA
hsa-miR-195	TAGCAGCACAGAAATATTGGCA
hsa-miR-196a	TAGGTAGTTTCATGTTGTTGGG
hsa-miR-196a	TAGGTAGTTTCATGTTGTTGGGA
hsa-miR-197	TTCACCACCTTCTCCACCCAGC
hsa-miR-197	TTCACCACCTTCTCCACCCAG
hsa-miR-197	TTCACCACCTTCTCCACCCAGA
hsa-miR-197	TTCACCACCTTCTCCACCCAGT
hsa-miR-199a-3p	ACAGTAGTCTGCACATTGGTT
hsa-miR-199a-3p	ACAGTAGTCTGCACATTGGTTA
hsa-miR-199a-3p	ACAGTAGTCTGCACATTGGTTT
hsa-miR-199a-3p	ACAGTAGTCTGCACATTGGTTAA
hsa-miR-199b-3p	ACAGTAGTCTGCACATTGGTT
hsa-miR-199b-3p	ACAGTAGTCTGCACATTGGTTA
hsa-miR-199b-3p	ACAGTAGTCTGCACATTGGTTT
hsa-miR-199b-3p	ACAGTAGTCTGCACATTGGTTAA
hsa-miR-19a	TGTGCAAATCTATGCAAAACTGA
hsa-miR-19b	TGTGCAAATCCATGCAAAACTGA
hsa-miR-19b	TGTGCAAATCCATGCAAAACTG
hsa-miR-19b	TGTGCAAATCCATGCAAAACTGT
hsa-miR-200a	TAACACTGTCTGGTAACGATGTT
hsa-miR-200b	TAATACTGCCTGGTAATGATGAC
hsa-miR-200c	TAATACTGCCGGGTAATGATGGA
hsa-miR-200c	TAATACTGCCGGGTAATGATGG
hsa-miR-20a	TAAAGTGCTTATAGTGCAGGTAG
hsa-miR-20a	TAAAGTGCTTATAGTGCAGGTA
hsa-miR-20a	TAAAGTGCTTATAGTGCAGGTAA
hsa-miR-20a	TAAAGTGCTTATAGTGCAGGTAT
hsa-miR-20a*	ACTGCATTATGAGCACTTAAAGT
hsa-miR-20b	CAAAGTGCTCATAGTGCAGGTAG
hsa-miR-20b	CAAAGTGCTCATAGTGCAGGTA

hsa-miR-20b	CAAAGTGCTCATAGTGCAGGTAA
hsa-miR-20b	CAAAGTGCTCATAGTGCAGGTAT
hsa-miR-20b*	ACTGTAGTATGGGCACTTCCA
hsa-miR-20b*	ACTGTAGTATGGGCACTTCCAGT
hsa-miR-20b*	ACTGTAGTATGGGCACTTCCAG
hsa-miR-21	TAGCTTATCAGACTGATGTTGAC
hsa-miR-21	TAGCTTATCAGACTGATGTTGA
hsa-miR-21	TAGCTTATCAGACTGATGTTG
hsa-miR-21	TAGCTTATCAGACTGATGTTGAA
hsa-miR-21	TAGCTTATCAGACTGATGTTGAT
hsa-miR-21	TAGCTTATCAGACTGATGTTGAG
hsa-miR-21	TAGCTTATCAGACTGATGTTGT
hsa-miR-21	TAGCTTATCAGACTGATGTTGCC
hsa-miR-21	TAGCTTATCAGACTGATGTTGC
hsa-miR-21	TAGCTTATCAGACTGATGTTGTC
hsa-miR-21	TAGCTTATCAGACTGATGTTGGC
hsa-miR-21	TAGCTTATCAGACTGATGTTGG
hsa-miR-21	TAGCTTATCAGACTGATGTTGTT
hsa-miR-21	TAGCTTATCAGACTGATGTTGCA
hsa-miR-21*	CAACACCAGTCGATGGGCTGTC
hsa-miR-21*	CAACACCAGTCGATGGGCTGT
hsa-miR-21*	CAACACCAGTCGATGGGCTG
hsa-miR-21*	CAACACCAGTCGATGGGCTGTA
hsa-miR-21*	CAACACCAGTCGATGGGCTGTAA
hsa-miR-21*	CAACACCAGTCGATGGGCTGTCA
hsa-miR-21*	CAACACCAGTCGATGGGCTGGC
hsa-miR-21*	CAACACCAGTCGATGGGCTGTT
hsa-miR-21*	CAACACCAGTCGATGGGCTGTCT
hsa-miR-21*	CAACACCAGTCGATGGGCTGG
hsa-miR-21*	CAACACCAGTCGATGGGCTGTAT
hsa-miR-21*	CAACACCAGTCGATGGGCTGTAG
hsa-miR-21*	CAACACCAGTCGATGGGCTGA
hsa-miR-21*	CAACACCAGTCGATGGGCTGGA
hsa-miR-21*	CAACACCAGTCGATGGGCTGTTA
hsa-miR-210	CTGTGCGTGTGACAGCGGCTGA
hsa-miR-210	CTGTGCGTGTGACAGCGGCTGAA
hsa-miR-210	CTGTGCGTGTGACAGCGGCTGT
hsa-miR-210	CTGTGCGTGTGACAGCGGCTG
hsa-miR-210	CTGTGCGTGTGACAGCGGCTGAT
hsa-miR-210	CTGTGCGTGTGACAGCGGCTGG
hsa-miR-210	CTGTGCGTGTGACAGCGGCTGTA
hsa-miR-210	CTGTGCGTGTGACAGCGGCTGGA
hsa-miR-210	CTGTGCGTGTGACAGCGGCTGAG
hsa-miR-210	CTGTGCGTGTGACAGCGGCTGC
hsa-miR-210	CTGTGCGTGTGACAGCGGCTGGT
hsa-miR-210	CTGTGCGTGTGACAGCGGCTGTT
hsa-miR-210	CTGTGCGTGTGACAGCGGCTGAC

hsa-miR-2110	TTGGGGAAACGGCCGCTGAGTGA
hsa-miR-2110	TTGGGGAAACGGCCGCTGAGTG
hsa-miR-2110	TTGGGGAAACGGCCGCTGAGT
hsa-miR-212	TAACAGTCTCCAGTCACGGCCA
hsa-miR-212	TAACAGTCTCCAGTCACGGCCAT
hsa-miR-215	ATGACCTATGAATTGACAGACA
hsa-miR-215	ATGACCTATGAATTGACAGAC
hsa-miR-219-1-3p	AGAGTTGAGTCTGGACGTCCCG
hsa-miR-219-5p	TGATTGTCCAAACGCAATTCTCG
hsa-miR-22	AAGCTGCCAGTTGAAGAACTGT
hsa-miR-22	AAGCTGCCAGTTGAAGAACTG
hsa-miR-22	AAGCTGCCAGTTGAAGAACTGTT
hsa-miR-22	AAGCTGCCAGTTGAAGAACTGTA
hsa-miR-22	AAGCTGCCAGTTGAAGAACTGA
hsa-miR-22	AAGCTGCCAGTTGAAGAACTGG
hsa-miR-22*	AGTTCTTCAGTGGCAAGCTTT
hsa-miR-22*	AGTTCTTCAGTGGCAAGCTTTA
hsa-miR-22*	AGTTCTTCAGTGGCAAGCTTTT
hsa-miR-221	AGCTACATTGTCTGCTGGGTTT
hsa-miR-221	AGCTACATTGTCTGCTGGGTTTA
hsa-miR-221	AGCTACATTGTCTGCTGGGTTTT
hsa-miR-221	AGCTACATTGTCTGCTGGGTTTC
hsa-miR-221	AGCTACATTGTCTGCTGGGTTTG
hsa-miR-221*	ACCTGGCATACAATGTAGATTT
hsa-miR-221*	ACCTGGCATACAATGTAGATT
hsa-miR-221*	ACCTGGCATACAATGTAGATTTC
hsa-miR-221*	ACCTGGCATACAATGTAGATTTT
hsa-miR-221*	ACCTGGCATACAATGTAGATTTA
hsa-miR-221*	ACCTGGCATACAATGTAGATTA
hsa-miR-221*	ACCTGGCATACAATGTAGATTG
hsa-miR-222	AGCTACATCTGGCTACTGGGTCT
hsa-miR-222	AGCTACATCTGGCTACTGGGT
hsa-miR-222	AGCTACATCTGGCTACTGGGTC
hsa-miR-222	AGCTACATCTGGCTACTGGGTCA
hsa-miR-222	AGCTACATCTGGCTACTGGGTAA
hsa-miR-222	AGCTACATCTGGCTACTGGGTA
hsa-miR-222	AGCTACATCTGGCTACTGGGGCT
hsa-miR-222	AGCTACATCTGGCTACTGGG
hsa-miR-222	AGCTACATCTGGCTACTGGGTAT
hsa-miR-222	AGCTACATCTGGCTACTGGGTT
hsa-miR-222	AGCTACATCTGGCTACTGGGG
hsa-miR-222	AGCTACATCTGGCTACTGGGTTT
hsa-miR-222	AGCTACATCTGGCTACTGGGGC
hsa-miR-222	AGCTACATCTGGCTACTGGGTCG
hsa-miR-222	AGCTACATCTGGCTACTGGGA
hsa-miR-222	AGCTACATCTGGCTACTGGGTTA
hsa-miR-222	AGCTACATCTGGCTACTGGGCCT

hsa-miR-222	AGCTACATCTGGCTACTGGGGCA
hsa-miR-222	AGCTACATCTGGCTACTGGGAA
hsa-miR-222	AGCTACATCTGGCTACTGGGGAA
hsa-miR-222	AGCTACATCTGGCTACTGGGACT
hsa-miR-222	AGCTACATCTGGCTACTGGGTCC
hsa-miR-222	AGCTACATCTGGCTACTGGGTAG
hsa-miR-222	AGCTACATCTGGCTACTGGGGA
hsa-miR-222	AGCTACATCTGGCTACTGGGAAA
hsa-miR-222	AGCTACATCTGGCTACTGGGC
hsa-miR-222	AGCTACATCTGGCTACTGGGCC
hsa-miR-222	AGCTACATCTGGCTACTGGGGAT
hsa-miR-223	TGTCAGTTTGTCAAATACCCCA
hsa-miR-223	TGTCAGTTTGTCAAATACCCCAA
hsa-miR-223	TGTCAGTTTGTCAAATACCCCAT
hsa-miR-223*	CGTGTATTTGACAAGCTGAGTTG
hsa-miR-223*	CGTGTATTTGACAAGCTGAGTT
hsa-miR-223*	CGTGTATTTGACAAGCTGAGT
hsa-miR-2355-3p	ATTGTCCTTGCTGTTTGGAGAT
hsa-miR-2355-5p	ATCCCCAGATACAATGGACAAT
hsa-miR-23a	ATCACATTGCCAGGGATTTCCA
hsa-miR-23a	ATCACATTGCCAGGGATTTC
hsa-miR-23a	ATCACATTGCCAGGGATTTCCAA
hsa-miR-23a	ATCACATTGCCAGGGATTTCC
hsa-miR-23a	ATCACATTGCCAGGGATTTCCAT
hsa-miR-23a	ATCACATTGCCAGGGATTTCA
hsa-miR-23a	ATCACATTGCCAGGGATTTCT
hsa-miR-23a	ATCACATTGCCAGGGATTTCCAG
hsa-miR-23a	ATCACATTGCCAGGGATTTCAA
hsa-miR-23a	ATCACATTGCCAGGGATTTCCT
hsa-miR-23a	ATCACATTGCCAGGGATTTCG
hsa-miR-23a*	GGGGTTCCTGGGGATGGGATTT
hsa-miR-23a*	GGGGTTCCTGGGGATGGGATT
hsa-miR-23b	ATCACATTGCCAGGGATTACCA
hsa-miR-23b	ATCACATTGCCAGGGATTACCAT
hsa-miR-23b	ATCACATTGCCAGGGATTACC
hsa-miR-23b	ATCACATTGCCAGGGATTAC
hsa-miR-23b	ATCACATTGCCAGGGATTACCAC
hsa-miR-23b	ATCACATTGCCAGGGATTACCAA
hsa-miR-23b*	TGGGTTCCTGGCATGCTGATTT
hsa-miR-23b*	TGGGTTCCTGGCATGCTGATT
hsa-miR-24	TGGCTCAGTTCAGCAGGAACAGT
hsa-miR-24	TGGCTCAGTTCAGCAGGAACAG
hsa-miR-24	TGGCTCAGTTCAGCAGGAACA
hsa-miR-24	TGGCTCAGTTCAGCAGGAACAGA
hsa-miR-24	TGGCTCAGTTCAGCAGGAACAA
hsa-miR-24	TGGCTCAGTTCAGCAGGAACAT
hsa-miR-24	TGGCTCAGTTCAGCAGGAACATT

hsa-miR-25	CATTGCACTTGTCTCGGTCTGA
hsa-miR-25	CATTGCACTTGTCTCGGTCTG
hsa-miR-25	CATTGCACTTGTCTCGGTCTGT
hsa-miR-25	CATTGCACTTGTCTCGGTCTGAA
hsa-miR-25	CATTGCACTTGTCTCGGTCTGAT
hsa-miR-25	CATTGCACTTGTCTCGGTCTGTA
hsa-miR-25	CATTGCACTTGTCTCGGTCTGC
hsa-miR-25	CATTGCACTTGTCTCGGTCTGG
hsa-miR-25	CATTGCACTTGTCTCGGTCTGTT
hsa-miR-25	CATTGCACTTGTCTCGGTCTGAG
hsa-miR-25	CATTGCACTTGTCTCGGTCTGAC
hsa-miR-25*	AGGCGGAGACTTGGGCAATTGCT
hsa-miR-25*	AGGCGGAGACTTGGGCAATTG
hsa-miR-25*	AGGCGGAGACTTGGGCAATTGC
hsa-miR-25*	AGGCGGAGACTTGGGCAATT
hsa-miR-25*	AGGCGGAGACTTGGGCAATTGT
hsa-miR-25*	AGGCGGAGACTTGGGCAATTGA
hsa-miR-25*	AGGCGGAGACTTGGGCAATTT
hsa-miR-25*	AGGCGGAGACTTGGGCAATTGAT
hsa-miR-25*	AGGCGGAGACTTGGGCAATTGTT
hsa-miR-25*	AGGCGGAGACTTGGGCAATTGCA
hsa-miR-25*	AGGCGGAGACTTGGGCAATTTCT
hsa-miR-25*	AGGCGGAGACTTGGGCAATTA
hsa-miR-25*	AGGCGGAGACTTGGGCAATTGAA
hsa-miR-25*	AGGCGGAGACTTGGGCAATTGCG
hsa-miR-26a	TTCAAGTAATCCAGGATAGGCT
hsa-miR-26a	TTCAAGTAATCCAGGATAGGCTA
hsa-miR-26a	TTCAAGTAATCCAGGATAGGC
hsa-miR-26a	TTCAAGTAATCCAGGATAGGCTT
hsa-miR-26a	TTCAAGTAATCCAGGATAGGCA
hsa-miR-26a	TTCAAGTAATCCAGGATAGGCAA
hsa-miR-26a	TTCAAGTAATCCAGGATAGGCTG
hsa-miR-26a	TTCAAGTAATCCAGGATAGGCG
hsa-miR-26a	TTCAAGTAATCCAGGATAGGCAT
hsa-miR-26a-2*	CCTATTCTTGATTACTTGTTT
hsa-miR-26a-2*	CCTATTCTTGATTACTTGTTTC
hsa-miR-26b	TTCAAGTAATTCAGGATAGGTT
hsa-miR-26b	TTCAAGTAATTCAGGATAGGT
hsa-miR-26b	TTCAAGTAATTCAGGATAGGTTA
hsa-miR-26b	TTCAAGTAATTCAGGATAGGTTT
hsa-miR-26b	TTCAAGTAATTCAGGATAGGTA
hsa-miR-26b	TTCAAGTAATTCAGGATAGGTTG
hsa-miR-26b	TTCAAGTAATTCAGGATAGGGT
hsa-miR-26b	TTCAAGTAATTCAGGATAGG
hsa-miR-26b	TTCAAGTAATTCAGGATAGGTAA
hsa-miR-26b	TTCAAGTAATTCAGGATAGGCT
hsa-miR-26b	TTCAAGTAATTCAGGATAGGTG

hsa-miR-26b	TTCAAGTAATTCAGGATAGGAT
hsa-miR-26b	TTCAAGTAATTCAGGATAGGTAG
hsa-miR-26b	TTCAAGTAATTCAGGATAGGG
hsa-miR-26b	TTCAAGTAATTCAGGATAGGTAT
hsa-miR-26b	TTCAAGTAATTCAGGATAGGTC
hsa-miR-26b	TTCAAGTAATTCAGGATAGGGTA
hsa-miR-27a	TTCACAGTGGCTAAGTTCCG
hsa-miR-27a	TTCACAGTGGCTAAGTTCCGT
hsa-miR-27a	TTCACAGTGGCTAAGTTCCGA
hsa-miR-27a	TTCACAGTGGCTAAGTTCCGC
hsa-miR-27a	TTCACAGTGGCTAAGTTCCGTT
hsa-miR-27a	TTCACAGTGGCTAAGTTCCGAAA
hsa-miR-27a	TTCACAGTGGCTAAGTTCCGAA
hsa-miR-27a	TTCACAGTGGCTAAGTTCCGTTT
hsa-miR-27a	TTCACAGTGGCTAAGTTCCGTTA
hsa-miR-27a	TTCACAGTGGCTAAGTTCCGCA
hsa-miR-27a	TTCACAGTGGCTAAGTTCCGCAA
hsa-miR-27a	TTCACAGTGGCTAAGTTCCGAT
hsa-miR-27a	TTCACAGTGGCTAAGTTCCGCT
hsa-miR-27a	TTCACAGTGGCTAAGTTCCGTAA
hsa-miR-27a	TTCACAGTGGCTAAGTTCCGTA
hsa-miR-27a	TTCACAGTGGCTAAGTTCCGTAT
hsa-miR-27a*	AGGGCTTAGCTGCTTGTGAGCA
hsa-miR-27a*	AGGGCTTAGCTGCTTGTGAGC
hsa-miR-27b	TTCACAGTGGCTAAGTTCTG
hsa-miR-27b	TTCACAGTGGCTAAGTTCTGC
hsa-miR-27b	TTCACAGTGGCTAAGTTCTGCA
hsa-miR-27b	TTCACAGTGGCTAAGTTCTGA
hsa-miR-27b	TTCACAGTGGCTAAGTTCTGAA
hsa-miR-27b	TTCACAGTGGCTAAGTTCTGCAA
hsa-miR-27b	TTCACAGTGGCTAAGTTCTGCAT
hsa-miR-27b	TTCACAGTGGCTAAGTTCTGTT
hsa-miR-27b	TTCACAGTGGCTAAGTTCTGAAA
hsa-miR-27b	TTCACAGTGGCTAAGTTCTGCT
hsa-miR-27b	TTCACAGTGGCTAAGTTCTGT
hsa-miR-27b*	AGAGCTTAGCTGATTGGTGAACA
hsa-miR-27b*	AGAGCTTAGCTGATTGGTGAAC
hsa-miR-27b*	AGAGCTTAGCTGATTGGTGAA
hsa-miR-28-3p	CACTAGATTGTGAGCTCCTGGA
hsa-miR-28-3p	CACTAGATTGTGAGCTCCTGGAA
hsa-miR-28-5p	AAGGAGCTCACAGTCTATTGAG
hsa-miR-296-5p	AGGGCCCCCCCTCAATCCTGT
hsa-miR-29a	TAGCACCATCTGAAATCGGTTA
hsa-miR-29a	TAGCACCATCTGAAATCGGTT
hsa-miR-29a	TAGCACCATCTGAAATCGGTTAA
hsa-miR-29a	TAGCACCATCTGAAATCGGTTT
hsa-miR-29a	TAGCACCATCTGAAATCGGTTAT

hsa-miR-29a	TAGCACCATCTGAAATCGGTTC
hsa-miR-29a	TAGCACCATCTGAAATCGGTTTA
hsa-miR-29a	TAGCACCATCTGAAATCGGTTAG
hsa-miR-29a	TAGCACCATCTGAAATCGGTTTT
hsa-miR-29a*	ACTGATTTCTTTTGGTGTTCAGA
hsa-miR-29a*	ACTGATTTCTTTTGGTGTTCAG
hsa-miR-29a*	ACTGATTTCTTTTGGTGTTCA
hsa-miR-29b	TAGCACCATTTGAAATCAGTGTT
hsa-miR-29b	TAGCACCATTTGAAATCAGTGT
hsa-miR-29b	TAGCACCATTTGAAATCAGTGTA
hsa-miR-29b-1*	GCTGGTTTCATATGGTGGTTTAG
hsa-miR-29b-2*	CTGGTTTCACATGGTGGCTTAGA
hsa-miR-29b-2*	CTGGTTTCACATGGTGGCTTA
hsa-miR-29c	TAGCACCATTTGAAATCGGTTA
hsa-miR-29c	TAGCACCATTTGAAATCGGTT
hsa-miR-29c	TAGCACCATTTGAAATCGGTTT
hsa-miR-29c	TAGCACCATTTGAAATCGGTTAA
hsa-miR-29c	TAGCACCATTTGAAATCGGTTAT
hsa-miR-29c*	TGACCGATTTCTCCTGGTGTT
hsa-miR-30a	TGTAAACATCCTCGACTGGAAGC
hsa-miR-30a	TGTAAACATCCTCGACTGGAAG
hsa-miR-30a	TGTAAACATCCTCGACTGGAAGA
hsa-miR-30a*	CTTTCAGTCGGATGTTTGCAGC
hsa-miR-30a*	CTTTCAGTCGGATGTTTGCAGT
hsa-miR-30b	TGTAAACATCCTACACTCAGCT
hsa-miR-30b	TGTAAACATCCTACACTCAGC
hsa-miR-30b	TGTAAACATCCTACACTCAGCTA
hsa-miR-30b*	CTGGGAGGTGGATGTTTACTT
hsa-miR-30b*	CTGGGAGGTGGATGTTTACTTC
hsa-miR-30b*	CTGGGAGGTGGATGTTTACTTT
hsa-miR-30b*	CTGGGAGGTGGATGTTTACTTA
hsa-miR-30c	TGTAAACATCCTACACTCTCAGC
hsa-miR-30c	TGTAAACATCCTACACTCTCAG
hsa-miR-30c	TGTAAACATCCTACACTCTCAGA
hsa-miR-30c-1*	CTGGGAGAGGGTTGTTTACTC
hsa-miR-30c-1*	CTGGGAGAGGGTTGTTTACTCC
hsa-miR-30c-1*	CTGGGAGAGGGTTGTTTACTCT
hsa-miR-30d	TGTAAACATCCCCGACTGGAAGC
hsa-miR-30d	TGTAAACATCCCCGACTGGAAG
hsa-miR-30d	TGTAAACATCCCCGACTGGAAGA
hsa-miR-30d	TGTAAACATCCCCGACTGGAA
hsa-miR-30d	TGTAAACATCCCCGACTGGAAA
hsa-miR-30d	TGTAAACATCCCCGACTGGAAGT
hsa-miR-30d	TGTAAACATCCCCGACTGGAAAA
hsa-miR-30d*	CTTTCAGTCAGATGTTTGCTGC
hsa-miR-30d*	CTTTCAGTCAGATGTTTGCTGCT
hsa-miR-30e	TGTAAACATCCTTGACTGGAAGC

hsa-miR-30e	TGTAAACATCCTTGACTGGAAG
hsa-miR-30e	TGTAAACATCCTTGACTGGAAGA
hsa-miR-30e	TGTAAACATCCTTGACTGGAA
hsa-miR-30e*	CTTTCAGTCGGATGTTTACAGT
hsa-miR-30e*	CTTTCAGTCGGATGTTTACAGC
hsa-miR-30e*	CTTTCAGTCGGATGTTTACAG
hsa-miR-30e*	CTTTCAGTCGGATGTTTACAGTT
hsa-miR-30e*	CTTTCAGTCGGATGTTTACAGCA
hsa-miR-30e*	CTTTCAGTCGGATGTTTACAGA
hsa-miR-30e*	CTTTCAGTCGGATGTTTACAGTA
hsa-miR-30e*	CTTTCAGTCGGATGTTTACAGCT
hsa-miR-30e*	CTTTCAGTCGGATGTTTACAGG
hsa-miR-30e*	CTTTCAGTCGGATGTTTACAGAA
hsa-miR-30e*	CTTTCAGTCGGATGTTTACAGAT
hsa-miR-30e*	CTTTCAGTCGGATGTTTACAGCC
hsa-miR-30e*	CTTTCAGTCGGATGTTTACAGGT
hsa-miR-3121	TAAATAGAGTAGGCAAAGGACA
hsa-miR-3127	ATCAGGGCTTGTGGAATGGGAAG
hsa-miR-3127	ATCAGGGCTTGTGGAATGGGAA
hsa-miR-3130-3p	GCTGCACCGGAGACTGGGTAA
hsa-miR-3130-3p	GCTGCACCGGAGACTGGGTAAG
hsa-miR-3136	CTGACTGAATAGGTAGGGTCAT
hsa-miR-3140	AGCTTTTGGGAATTCAGGTAG
hsa-miR-3140	AGCTTTTGGGAATTCAGGTAGT
hsa-miR-3141	GAGGGCGGGTGGAGGAGGAA
hsa-miR-3149	TTTGTATGGATATGTGTGTGTAT
hsa-miR-3153	GGGGAAAGCGAGTAGGGACATT
hsa-miR-3158	AAGGGCTTCCTCTCTGCAGGAC
hsa-miR-3158	AAGGGCTTCCTCTCTGCAGGACA
hsa-miR-3158	AAGGGCTTCCTCTCTGCAGGA
hsa-miR-3164	TGTGACTTTAAGGGAAATGGCG
hsa-miR-3165	AGGTGGATGCAATGTGACCTCA
hsa-miR-3179	AGAAGGGGTGAAATTTAAACGT
hsa-miR-3179	AGAAGGGGTGAAATTTAAACG
hsa-miR-3182	GCTTCTGTAGTGTAGTGGTTAT
hsa-miR-3182	GCTTCTGTAGTGTAGTGG
hsa-miR-3182	GCTTCTGTAGTGTAGTGGTTATC
hsa-miR-3182	GCTTCTGTAGTGTAGTGGT
hsa-miR-3182	GCTTCTGTAGTGTAGTGGTT
hsa-miR-3182	GCTTCTGTAGTGTAGTGGTTA
hsa-miR-3182	GCTTCTGTAGTGTAGTGGA
hsa-miR-3182	GCTTCTGTAGTGTAGTGGAA
hsa-miR-3192	TCTGGGAGGTTGTAGCAGTGGAA
hsa-miR-32	TATTGCACATTACTAAGTTGCAT
hsa-miR-32	TATTGCACATTACTAAGTTGCA
hsa-miR-32*	CAATTTAGTGTGTGTGATATT
hsa-miR-320a	AAAAGCTGGGTTGAGAGGGCGA

hsa-miR-320a	AAAAGCTGGGTTGAGAGGGCGAA
hsa-miR-320a	AAAAGCTGGGTTGAGAGGGCGAT
hsa-miR-320a	AAAAGCTGGGTTGAGAGGGCGT
hsa-miR-320a	AAAAGCTGGGTTGAGAGGGCGTT
hsa-miR-320a	AAAAGCTGGGTTGAGAGGGCG
hsa-miR-320a	AAAAGCTGGGTTGAGAGGGCGTA
hsa-miR-320a	AAAAGCTGGGTTGAGAGGGCGAG
hsa-miR-320a	AAAAGCTGGGTTGAGAGGGCGC
hsa-miR-320a	AAAAGCTGGGTTGAGAGGGCGAC
hsa-miR-320a	AAAAGCTGGGTTGAGAGGGCGG
hsa-miR-320a	AAAAGCTGGGTTGAGAGGGCGCT
hsa-miR-320a	AAAAGCTGGGTTGAGAGGGCGCA
hsa-miR-320a	AAAAGCTGGGTTGAGAGGGCGGT
hsa-miR-320a	AAAAGCTGGGTTGAGAGGGCGGA
hsa-miR-320a	AAAAGCTGGGTTGAGAGGGCGTG
hsa-miR-320a	AAAAGCTGGGTTGAGAGGGCGTC
hsa-miR-320a	AAAAGCTGGGTTGAGAGGGCGCC
hsa-miR-320b	AAAAGCTGGGTTGAGAGGGCAA
hsa-miR-320b	AAAAGCTGGGTTGAGAGGGCAT
hsa-miR-320b	AAAAGCTGGGTTGAGAGGGCA
hsa-miR-320b	AAAAGCTGGGTTGAGAGGGCAAA
hsa-miR-320b	AAAAGCTGGGTTGAGAGGGCAAT
hsa-miR-320b	AAAAGCTGGGTTGAGAGGGCATT
hsa-miR-320b	AAAAGCTGGGTTGAGAGGGCATA
hsa-miR-320b	AAAAGCTGGGTTGAGAGGGCAG
hsa-miR-320c	AAAAGCTGGGTTGAGAGGGCGA
hsa-miR-320c	AAAAGCTGGGTTGAGAGGGCGAA
hsa-miR-320c	AAAAGCTGGGTTGAGAGGGCGAT
hsa-miR-320c	AAAAGCTGGGTTGAGAGGGCGT
hsa-miR-320c	AAAAGCTGGGTTGAGAGGGCGTT
hsa-miR-320c	AAAAGCTGGGTTGAGAGGGCG
hsa-miR-320c	AAAAGCTGGGTTGAGAGGGCGTA
hsa-miR-320c	AAAAGCTGGGTTGAGAGGGCGAG
hsa-miR-320c	AAAAGCTGGGTTGAGAGGGAGA
hsa-miR-320c	AAAAGCTGGGTTGAGAGGGCAA
hsa-miR-320c	AAAAGCTGGGTTGAGAGGGCGC
hsa-miR-320c	AAAAGCTGGGTTGAGAGGGC
hsa-miR-320c	AAAAGCTGGGTTGAGAGGGAGAA
hsa-miR-320c	AAAAGCTGGGTTGAGAGGGCGAC
hsa-miR-320c	AAAAGCTGGGTTGAGAGGGCAT
hsa-miR-320c	AAAAGCTGGGTTGAGAGGGCGG
hsa-miR-320c	AAAAGCTGGGTTGAGAGGGGGA
hsa-miR-320c	AAAAGCTGGGTTGAGAGGGCA
hsa-miR-320c	AAAAGCTGGGTTGAGAGGGCTA
hsa-miR-320c	AAAAGCTGGGTTGAGAGGG
hsa-miR-320c	AAAAGCTGGGTTGAGAGGGAGAT
hsa-miR-320c	AAAAGCTGGGTTGAGAGGGTGA

hsa-miR-320c	AAAAGCTGGGTTGAGAGGGCAAA
hsa-miR-320c	AAAAGCTGGGTTGAGAGGGCGCT
hsa-miR-320c	AAAAGCTGGGTTGAGAGGGCGCA
hsa-miR-320c	AAAAGCTGGGTTGAGAGGGCTAA
hsa-miR-320c	AAAAGCTGGGTTGAGAGGGCGGT
hsa-miR-320c	AAAAGCTGGGTTGAGAGGGCAAT
hsa-miR-320c	AAAAGCTGGGTTGAGAGGGCTAT
hsa-miR-320c	AAAAGCTGGGTTGAGAGGGGGAA
hsa-miR-320c	AAAAGCTGGGTTGAGAGGGAGT
hsa-miR-320c	AAAAGCTGGGTTGAGAGGGTGAA
hsa-miR-320c	AAAAGCTGGGTTGAGAGGGCGGA
hsa-miR-320c	AAAAGCTGGGTTGAGAGGGA
hsa-miR-320c	AAAAGCTGGGTTGAGAGGGCATT
hsa-miR-320c	AAAAGCTGGGTTGAGAGGGGGAT
hsa-miR-320c	AAAAGCTGGGTTGAGAGGGCTT
hsa-miR-320c	AAAAGCTGGGTTGAGAGGGCGTG
hsa-miR-320c	AAAAGCTGGGTTGAGAGGGCGTC
hsa-miR-320c	AAAAGCTGGGTTGAGAGGGAAA
hsa-miR-320c	AAAAGCTGGGTTGAGAGGGAA
hsa-miR-320c	AAAAGCTGGGTTGAGAGGGAGTT
hsa-miR-320c	AAAAGCTGGGTTGAGAGGGGGT
hsa-miR-320c	AAAAGCTGGGTTGAGAGGGAG
hsa-miR-320c	AAAAGCTGGGTTGAGAGGGTTT
hsa-miR-320c	AAAAGCTGGGTTGAGAGGGTGAT
hsa-miR-320c	AAAAGCTGGGTTGAGAGGGTGT
hsa-miR-320c	AAAAGCTGGGTTGAGAGGGAT
hsa-miR-320c	AAAAGCTGGGTTGAGAGGGCCA
hsa-miR-320c	AAAAGCTGGGTTGAGAGGGT
hsa-miR-320c	AAAAGCTGGGTTGAGAGGGCT
hsa-miR-320c	AAAAGCTGGGTTGAGAGGGCATA
hsa-miR-320c	AAAAGCTGGGTTGAGAGGGGGTT
hsa-miR-320c	AAAAGCTGGGTTGAGAGGGCTTT
hsa-miR-320c	AAAAGCTGGGTTGAGAGGGTAA
hsa-miR-320c	AAAAGCTGGGTTGAGAGGGGG
hsa-miR-320c	AAAAGCTGGGTTGAGAGGGTT
hsa-miR-320c	AAAAGCTGGGTTGAGAGGGAGAG
hsa-miR-320c	AAAAGCTGGGTTGAGAGGGAAT
hsa-miR-320c	AAAAGCTGGGTTGAGAGGGAGTA
hsa-miR-320c	AAAAGCTGGGTTGAGAGGGTTA
hsa-miR-320c	AAAAGCTGGGTTGAGAGGGTGTT
hsa-miR-320c	AAAAGCTGGGTTGAGAGGGAAAA
hsa-miR-320c	AAAAGCTGGGTTGAGAGGGTTTT
hsa-miR-320c	AAAAGCTGGGTTGAGAGGGAGGA
hsa-miR-320c	AAAAGCTGGGTTGAGAGGGCGCC
hsa-miR-320c	AAAAGCTGGGTTGAGAGGGCAG
hsa-miR-320c	AAAAGCTGGGTTGAGAGGGAGG
hsa-miR-320c	AAAAGCTGGGTTGAGAGGGTG

hsa-miR-320c	AAAAGCTGGGTTGAGAGGGCTTA
hsa-miR-320c	AAAAGCTGGGTTGAGAGGGAGGT
hsa-miR-320c	AAAAGCTGGGTTGAGAGGGTTTA
hsa-miR-320c	AAAAGCTGGGTTGAGAGGGCCAA
hsa-miR-320d	AAAAGCTGGGTTGAGAGGGCGA
hsa-miR-320d	AAAAGCTGGGTTGAGAGGGCGAA
hsa-miR-320d	AAAAGCTGGGTTGAGAGGGCGAT
hsa-miR-320d	AAAAGCTGGGTTGAGAGGGCGT
hsa-miR-320d	AAAAGCTGGGTTGAGAGGGCGTT
hsa-miR-320d	AAAAGCTGGGTTGAGAGGGCG
hsa-miR-320d	AAAAGCTGGGTTGAGAGGGCGTA
hsa-miR-320d	AAAAGCTGGGTTGAGAGGGCGAG
hsa-miR-320d	AAAAGCTGGGTTGAGAGGGAGA
hsa-miR-320d	AAAAGCTGGGTTGAGAGGGCAA
hsa-miR-320d	AAAAGCTGGGTTGAGAGGGCGC
hsa-miR-320d	AAAAGCTGGGTTGAGAGGGC
hsa-miR-320d	AAAAGCTGGGTTGAGAGGGAGAA
hsa-miR-320d	AAAAGCTGGGTTGAGAGGGCGAC
hsa-miR-320d	AAAAGCTGGGTTGAGAGGGCAT
hsa-miR-320d	AAAAGCTGGGTTGAGAGGGCGG
hsa-miR-320d	AAAAGCTGGGTTGAGAGGGGGA
hsa-miR-320d	AAAAGCTGGGTTGAGAGGCCGA
hsa-miR-320d	AAAAGCTGGGTTGAGAGGGCA
hsa-miR-320d	AAAAGCTGGGTTGAGAGGGCTA
hsa-miR-320d	AAAAGCTGGGTTGAGAGG
hsa-miR-320d	AAAAGCTGGGTTGAGAGGG
hsa-miR-320d	AAAAGCTGGGTTGAGAGGGAGAT
hsa-miR-320d	AAAAGCTGGGTTGAGAGGTCGA
hsa-miR-320d	AAAAGCTGGGTTGAGAGGACGA
hsa-miR-320d	AAAAGCTGGGTTGAGAGGGTGA
hsa-miR-320d	AAAAGCTGGGTTGAGAGGGCAAA
hsa-miR-320d	AAAAGCTGGGTTGAGAGGGCGCT
hsa-miR-320d	AAAAGCTGGGTTGAGAGGGCGCA
hsa-miR-320d	AAAAGCTGGGTTGAGAGGGCTAA
hsa-miR-320d	AAAAGCTGGGTTGAGAGGGCGGT
hsa-miR-320d	AAAAGCTGGGTTGAGAGGGCAAT
hsa-miR-320d	AAAAGCTGGGTTGAGAGGGCTAT
hsa-miR-320d	AAAAGCTGGGTTGAGAGGGGGAA
hsa-miR-320d	AAAAGCTGGGTTGAGAGGGAGT
hsa-miR-320d	AAAAGCTGGGTTGAGAGGGTGAA
hsa-miR-320d	AAAAGCTGGGTTGAGAGGGCGGA
hsa-miR-320d	AAAAGCTGGGTTGAGAGGGA
hsa-miR-320d	AAAAGCTGGGTTGAGAGGCCGAA
hsa-miR-320d	AAAAGCTGGGTTGAGAGGGCATT
hsa-miR-320d	AAAAGCTGGGTTGAGAGGTCGAA
hsa-miR-320d	AAAAGCTGGGTTGAGAGGGGGAT
hsa-miR-320d	AAAAGCTGGGTTGAGAGGGCTT

hsa-miR-320d	AAAAGCTGGGTTGAGAGGGCGTG
hsa-miR-320d	AAAAGCTGGGTTGAGAGGGCGTC
hsa-miR-320d	AAAAGCTGGGTTGAGAGGGAAA
hsa-miR-320d	AAAAGCTGGGTTGAGAGGACGAA
hsa-miR-320d	AAAAGCTGGGTTGAGAGGGAA
hsa-miR-320d	AAAAGCTGGGTTGAGAGGGAGTT
hsa-miR-320d	AAAAGCTGGGTTGAGAGGGGGT
hsa-miR-320d	AAAAGCTGGGTTGAGAGGGAG
hsa-miR-320d	AAAAGCTGGGTTGAGAGGGTTT
hsa-miR-320d	AAAAGCTGGGTTGAGAGGGTGAT
hsa-miR-320d	AAAAGCTGGGTTGAGAGGTCGAT
hsa-miR-320d	AAAAGCTGGGTTGAGAGGCCGAT
hsa-miR-320d	AAAAGCTGGGTTGAGAGGGTGT
hsa-miR-320d	AAAAGCTGGGTTGAGAGGACGAT
hsa-miR-320d	AAAAGCTGGGTTGAGAGGCCGT
hsa-miR-320d	AAAAGCTGGGTTGAGAGGGAT
hsa-miR-320d	AAAAGCTGGGTTGAGAGGGCCA
hsa-miR-320d	AAAAGCTGGGTTGAGAGGGT
hsa-miR-320d	AAAAGCTGGGTTGAGAGGGCT
hsa-miR-320d	AAAAGCTGGGTTGAGAGGACGT
hsa-miR-320d	AAAAGCTGGGTTGAGAGGGCATA
hsa-miR-320d	AAAAGCTGGGTTGAGAGGCGA
hsa-miR-320d	AAAAGCTGGGTTGAGAGGGGGTT
hsa-miR-320d	AAAAGCTGGGTTGAGAGGGCTTT
hsa-miR-320d	AAAAGCTGGGTTGAGAGGTCGT
hsa-miR-320d	AAAAGCTGGGTTGAGAGGGTAA
hsa-miR-320d	AAAAGCTGGGTTGAGAGGCCG
hsa-miR-320d	AAAAGCTGGGTTGAGAGGGGG
hsa-miR-320d	AAAAGCTGGGTTGAGAGGGTT
hsa-miR-320d	AAAAGCTGGGTTGAGAGGGAGAG
hsa-miR-320d	AAAAGCTGGGTTGAGAGGCGAA
hsa-miR-320d	AAAAGCTGGGTTGAGAGGAA
hsa-miR-320d	AAAAGCTGGGTTGAGAGGTCTA
hsa-miR-320d	AAAAGCTGGGTTGAGAGGGAAT
hsa-miR-320d	AAAAGCTGGGTTGAGAGGGAGTA
hsa-miR-320d	AAAAGCTGGGTTGAGAGGGTTA
hsa-miR-320d	AAAAGCTGGGTTGAGAGGGTGTT
hsa-miR-320d	AAAAGCTGGGTTGAGAGGGAAAA
hsa-miR-320d	AAAAGCTGGGTTGAGAGGGTTTT
hsa-miR-320d	AAAAGCTGGGTTGAGAGGAAA
hsa-miR-320d	AAAAGCTGGGTTGAGAGGGAGGA
hsa-miR-320d	AAAAGCTGGGTTGAGAGGGCGCC
hsa-miR-320d	AAAAGCTGGGTTGAGAGGGCAG
hsa-miR-320d	AAAAGCTGGGTTGAGAGGCCGTT
hsa-miR-320d	AAAAGCTGGGTTGAGAGGA
hsa-miR-320d	AAAAGCTGGGTTGAGAGGACGTT
hsa-miR-320d	AAAAGCTGGGTTGAGAGGGAGG

hsa-miR-320d	AAAAGCTGGGTTGAGAGGACG
hsa-miR-320d	AAAAGCTGGGTTGAGAGGGTG
hsa-miR-320d	AAAAGCTGGGTTGAGAGGGCTTA
hsa-miR-320d	AAAAGCTGGGTTGAGAGGCGAT
hsa-miR-320d	AAAAGCTGGGTTGAGAGGGAGGT
hsa-miR-320d	AAAAGCTGGGTTGAGAGGTTT
hsa-miR-320d	AAAAGCTGGGTTGAGAGGGTTTA
hsa-miR-320d	AAAAGCTGGGTTGAGAGGGCCAA
hsa-miR-324-3p	ACTGCCCCAGGTGCTGCTGGT
hsa-miR-324-3p	ACTGCCCCAGGTGCTGCTGGTA
hsa-miR-324-3p	ACTGCCCCAGGTGCTGCTGGTAA
hsa-miR-324-3p	ACTGCCCCAGGTGCTGCTGGTAT
hsa-miR-324-3p	ACTGCCCCAGGTGCTGCTGGTT
hsa-miR-324-5p	CGCATCCCCTAGGGCATTGGTGT
hsa-miR-324-5p	CGCATCCCCTAGGGCATTGGTG
hsa-miR-324-5p	CGCATCCCCTAGGGCATTGGTGA
hsa-miR-328	CTGGCCCTCTCTGCCCTTCCGT
hsa-miR-328	CTGGCCCTCTCTGCCCTTCCGTT
hsa-miR-330-3p	GCAAAGCACACGGCCTGCAGAGA
hsa-miR-330-3p	GCAAAGCACACGGCCTGCAGAG
hsa-miR-330-3p	GCAAAGCACACGGCCTGCAGAGT
hsa-miR-331-3p	GCCCCTGGGCCTATCCTAGAA
hsa-miR-331-3p	GCCCCTGGGCCTATCCTAGA
hsa-miR-331-3p	GCCCCTGGGCCTATCCTAGAAA
hsa-miR-331-3p	GCCCCTGGGCCTATCCTAGAAT
hsa-miR-331-3p	GCCCCTGGGCCTATCCTAGAC
hsa-miR-331-3p	GCCCCTGGGCCTATCCTAGAT
hsa-miR-331-3p	GCCCCTGGGCCTATCCTAGACA
hsa-miR-331-3p	GCCCCTGGGCCTATCCTAGACT
hsa-miR-331-3p	GCCCCTGGGCCTATCCTAGAATT
hsa-miR-331-3p	GCCCCTGGGCCTATCCTAGAAAA
hsa-miR-331-3p	GCCCCTGGGCCTATCCTAGATT
hsa-miR-331-3p	GCCCCTGGGCCTATCCTAGAAAT
hsa-miR-335	TCAAGAGCAATAACGAAAAATG
hsa-miR-335	TCAAGAGCAATAACGAAAAATGT
hsa-miR-339-3p	TGAGCGCCTCGACGACAGAGCCG
hsa-miR-339-3p	TGAGCGCCTCGACGACAGAGCC
hsa-miR-339-3p	TGAGCGCCTCGACGACAGAGCCA
hsa-miR-339-3p	TGAGCGCCTCGACGACAGAGCCT
hsa-miR-339-5p	TCCCTGTCCTCCAGGAGCTCACG
hsa-miR-339-5p	TCCCTGTCCTCCAGGAGCTCAC
hsa-miR-339-5p	TCCCTGTCCTCCAGGAGCTCACA
hsa-miR-33a	GTGCATTGTAGTTGCATTGCA
hsa-miR-33a	GTGCATTGTAGTTGCATTGC
hsa-miR-33a	GTGCATTGTAGTTGCATTGCAA
hsa-miR-33a	GTGCATTGTAGTTGCATTGCAT
hsa-miR-33a	GTGCATTGTAGTTGCATTGCAAA

hsa-miR-33a*	CAATGTTTCCACAGTGCATCA
hsa-miR-33b	GTGCATTGCTGTTGCATTGCA
hsa-miR-33b	GTGCATTGCTGTTGCATTGC
hsa-miR-340	TTATAAAGCAATGAGACTGATT
hsa-miR-340	TTATAAAGCAATGAGACTGAT
hsa-miR-340	TTATAAAGCAATGAGACTGATA
hsa-miR-340	TTATAAAGCAATGAGACTGATTA
hsa-miR-342-3p	TCTCACACAGAAATCGCACCCGT
hsa-miR-342-3p	TCTCACACAGAAATCGCACCCG
hsa-miR-342-3p	TCTCACACAGAAATCGCACCCGA
hsa-miR-342-3p	TCTCACACAGAAATCGCACCCGG
hsa-miR-342-5p	AGGGGTGCTATCTGTGATTGAGG
hsa-miR-342-5p	AGGGGTGCTATCTGTGATTGAG
hsa-miR-342-5p	AGGGGTGCTATCTGTGATTGA
hsa-miR-342-5p	AGGGGTGCTATCTGTGATTGAGA
hsa-miR-345	GCTGACTCCTAGTCCAGGGCTCA
hsa-miR-345	GCTGACTCCTAGTCCAGGGCT
hsa-miR-345	GCTGACTCCTAGTCCAGGGCTCT
hsa-miR-345	GCTGACTCCTAGTCCAGGGCTAA
hsa-miR-345	GCTGACTCCTAGTCCAGGGCTA
hsa-miR-345	GCTGACTCCTAGTCCAGGGCTAT
hsa-miR-345	GCTGACTCCTAGTCCAGGGCTC
hsa-miR-345	GCTGACTCCTAGTCCAGGGCTT
hsa-miR-345	GCTGACTCCTAGTCCAGGGCTTT
hsa-miR-345	GCTGACTCCTAGTCCAGGGCTTA
hsa-miR-34a	TGGCAGTGTCTTAGCTGGTTGTT
hsa-miR-34a	TGGCAGTGTCTTAGCTGGTTGT
hsa-miR-34a	TGGCAGTGTCTTAGCTGGTTG
hsa-miR-34a	TGGCAGTGTCTTAGCTGGTTGTA
hsa-miR-34a*	CAATCAGCAAGTATACTGCCCTA
hsa-miR-34a*	CAATCAGCAAGTATACTGCCCTT
hsa-miR-34a*	CAATCAGCAAGTATACTGCCCT
hsa-miR-34c-5p	AGGCAGTGTAGTTAGCTGATTG
hsa-miR-34c-5p	AGGCAGTGTAGTTAGCTGATTGC
hsa-miR-3613-5p	TGTTGTACTTTTTTTTTTGTT
hsa-miR-361-3p	TCCCCCAGGTGTGATTCTGATTT
hsa-miR-361-3p	TCCCCCAGGTGTGATTCTGATT
hsa-miR-3614-3p	TAGCCTTCAGATCTTGGTGTTT
hsa-miR-3614-5p	CCACTTGGATCTGAAGGCTGCC
hsa-miR-3615	TCTCTCGGCTCCTCGCGGCT
hsa-miR-3615	TCTCTCGGCTCCTCGCGGCTCG
hsa-miR-3615	TCTCTCGGCTCCTCGCGGCTTT
hsa-miR-3615	TCTCTCGGCTCCTCGCGGCTC
hsa-miR-3615	TCTCTCGGCTCCTCGCGGCTT
hsa-miR-361-5p	TTATCAGAATCTCCAGGGGTAC
hsa-miR-361-5p	TTATCAGAATCTCCAGGGGTA
hsa-miR-361-5p	TTATCAGAATCTCCAGGGGTAT

hsa-miR-361-5p	TTATCAGAATCTCCAGGGGTAA
hsa-miR-361-5p	TTATCAGAATCTCCAGGGGTACT
hsa-miR-361-5p	TTATCAGAATCTCCAGGGGTACA
hsa-miR-361-5p	TTATCAGAATCTCCAGGGGTAAA
hsa-miR-361-5p	TTATCAGAATCTCCAGGGGTATA
hsa-miR-361-5p	TTATCAGAATCTCCAGGGGTATT
hsa-miR-362-3p	AACACACCTATTCAAGGATTCA
hsa-miR-362-3p	AACACACCTATTCAAGGATTC
hsa-miR-362-5p	AATCCTTGGAACCTAGGTGTGAG
hsa-miR-363	AATTGCACGGTATCCATCTGTA
hsa-miR-363	AATTGCACGGTATCCATCTGTAA
hsa-miR-363	AATTGCACGGTATCCATCTGT
hsa-miR-363	AATTGCACGGTATCCATCTGTAT
hsa-miR-363	AATTGCACGGTATCCATCTGTAG
hsa-miR-363	AATTGCACGGTATCCATCTGTT
hsa-miR-363	AATTGCACGGTATCCATCTGTTT
hsa-miR-363	AATTGCACGGTATCCATCTGTTA
hsa-miR-363*	CGGGTGGATCACGATGCAATTTT
hsa-miR-363*	CGGGTGGATCACGATGCAATTT
hsa-miR-363*	CGGGTGGATCACGATGCAATT
hsa-miR-365	TAATGCCCCTAAAAATCCTTAT
hsa-miR-365	TAATGCCCCTAAAAATCCTTA
hsa-miR-365	TAATGCCCCTAAAAATCCTTATT
hsa-miR-365	TAATGCCCCTAAAAATCCTTAA
hsa-miR-365	TAATGCCCCTAAAAATCCTTATA
hsa-miR-365	TAATGCCCCTAAAAATCCTTAC
hsa-miR-365	TAATGCCCCTAAAAATCCTTAAA
hsa-miR-365	TAATGCCCCTAAAAATCCTTAG
hsa-miR-3676	CCGTGTTTCCCCCACGCTTT
hsa-miR-3676	CCGTGTTTCCCCCACGCTTTT
hsa-miR-3676	CCGTGTTTCCCCCACGCTT
hsa-miR-3679-5p	TGAGGATATGGCAGGGAAGGGGA
hsa-miR-374a	TTATAATACAACCTGATAAGTG
hsa-miR-374a	TTATAATACAACCTGATAAGT
hsa-miR-374a	TTATAATACAACCTGATAAGTGA
hsa-miR-374a*	CTTATCAGATTGTATTGTAATT
hsa-miR-374a*	CTTATCAGATTGTATTGTAAT
hsa-miR-374a*	CTTATCAGATTGTATTGTAATTA
hsa-miR-374a*	CTTATCAGATTGTATTGTAATTT
hsa-miR-374a*	CTTATCAGATTGTATTGTAATA
hsa-miR-374b	ATATAATACAACCTGCTAAGTG
hsa-miR-374b	ATATAATACAACCTGCTAAGT
hsa-miR-374b	ATATAATACAACCTGCTAAGTGA
hsa-miR-374b	ATATAATACAACCTGCTAAGTGT
hsa-miR-374b	ATATAATACAACCTGCTAAGTT
hsa-miR-374b*	CTTAGCAGGTTGTATTATCATT
hsa-miR-374b*	CTTAGCAGGTTGTATTATCAT

hsa-miR-375	TTTGTTCGTTCGGCTCGCGTGA
hsa-miR-378	ACTGGACTTGGAGTCAGAAGG
hsa-miR-378	ACTGGACTTGGAGTCAGAAGGC
hsa-miR-378	ACTGGACTTGGAGTCAGAAGGCA
hsa-miR-378	ACTGGACTTGGAGTCAGAAG
hsa-miR-378	ACTGGACTTGGAGTCAGAAGGA
hsa-miR-378	ACTGGACTTGGAGTCAGAAGGCT
hsa-miR-378	ACTGGACTTGGAGTCAGAAGGAA
hsa-miR-378	ACTGGACTTGGAGTCAGAAGGT
hsa-miR-378	ACTGGACTTGGAGTCAGAAGA
hsa-miR-378	ACTGGACTTGGAGTCAGAAGGCG
hsa-miR-378	ACTGGACTTGGAGTCAGAAGAA
hsa-miR-378	ACTGGACTTGGAGTCAGAAGGTA
hsa-miR-378	ACTGGACTTGGAGTCAGAAGT
hsa-miR-378	ACTGGACTTGGAGTCAGAAGGAT
hsa-miR-378	ACTGGACTTGGAGTCAGAAGGTT
hsa-miR-378	ACTGGACTTGGAGTCAGAAGGAG
hsa-miR-378	ACTGGACTTGGAGTCAGAAGTC
hsa-miR-378	ACTGGACTTGGAGTCAGAAGGG
hsa-miR-378	ACTGGACTTGGAGTCAGAAGAAA
hsa-miR-378	ACTGGACTTGGAGTCAGAAGTCA
hsa-miR-378	ACTGGACTTGGAGTCAGAAGGCC
hsa-miR-378	ACTGGACTTGGAGTCAGAAGGGA
hsa-miR-378	ACTGGACTTGGAGTCAGAAGTA
hsa-miR-378	ACTGGACTTGGAGTCAGAAGAC
hsa-miR-378	ACTGGACTTGGAGTCAGAAGGAC
hsa-miR-378	ACTGGACTTGGAGTCAGAAGGTG
hsa-miR-378	ACTGGACTTGGAGTCAGAAGTT
hsa-miR-378	ACTGGACTTGGAGTCAGAAGCC
hsa-miR-378	ACTGGACTTGGAGTCAGAAGC
hsa-miR-378	ACTGGACTTGGAGTCAGAAGTCT
hsa-miR-378	ACTGGACTTGGAGTCAGAAGTAA
hsa-miR-378	ACTGGACTTGGAGTCAGAAGAT
hsa-miR-378	ACTGGACTTGGAGTCAGAAGGGT
hsa-miR-378*	CTCCTGACTCCAGGTCCTGTGT
hsa-miR-378b	ACTGGACTTGGAGGCAGAAGG
hsa-miR-378b	ACTGGACTTGGAGGCAGAAGGC
hsa-miR-378b	ACTGGACTTGGAGGCAGAAGGCA
hsa-miR-378b	ACTGGACTTGGAGGCAGAAG
hsa-miR-378b	ACTGGACTTGGAGGCAGAAGGA
hsa-miR-378b	ACTGGACTTGGAGGCAGAAGGCT
hsa-miR-378b	ACTGGACTTGGAGGCAGAAGGAA
hsa-miR-378b	ACTGGACTTGGAGGCAGAAGGT
hsa-miR-378b	ACTGGACTTGGAGGCAGA
hsa-miR-378b	ACTGGACTTGGAGGCAGAAGA
hsa-miR-3917	GCTCGGACTGAGCAGGTGGG
hsa-miR-3928	GGAGGAACCTTGGAGCTTCGGCA

hsa-miR-3928	GGAGGAACCTTGGAGCTTCGGT
hsa-miR-3928	GGAGGAACCTTGGAGCTTCGGC
hsa-miR-3928	GGAGGAACCTTGGAGCTTCGG
hsa-miR-3928	GGAGGAACCTTGGAGCTTCGGCT
hsa-miR-3928	GGAGGAACCTTGGAGCTTCGGTA
hsa-miR-3928	GGAGGAACCTTGGAGCTTCGGA
hsa-miR-3928	GGAGGAACCTTGGAGCTTCGGTT
hsa-miR-3934	TCAGGTGTGGAAACTGAGGCAG
hsa-miR-3934	TCAGGTGTGGAAACTGAGGCA
hsa-miR-3934	TCAGGTGTGGAAACTGAGGCAGG
hsa-miR-3934	TCAGGTGTGGAAACTGAGGCAGA
hsa-miR-3940	CAGCCCGGATCCCAGCCCACT
hsa-miR-421	ATCAACAGACATTAATTGGGCG
hsa-miR-421	ATCAACAGACATTAATTGGGCGT
hsa-miR-421	ATCAACAGACATTAATTGGGCGC
hsa-miR-421	ATCAACAGACATTAATTGGGCGA
hsa-miR-423-3p	AGCTCGGTCTGAGGCCCCTCAGT
hsa-miR-423-3p	AGCTCGGTCTGAGGCCCCTCAG
hsa-miR-423-3p	AGCTCGGTCTGAGGCCCCTCAGA
hsa-miR-423-3p	AGCTCGGTCTGAGGCCCCTCAGC
hsa-miR-423-3p	AGCTCGGTCTGAGGCCCCTCAGG
hsa-miR-423-5p	TGAGGGGCAGAGAGCGAGACTTT
hsa-miR-423-5p	TGAGGGGCAGAGAGCGAGACTT
hsa-miR-423-5p	TGAGGGGCAGAGAGCGAGACTTA
hsa-miR-423-5p	TGAGGGGCAGAGAGCGAGACTTG
hsa-miR-423-5p	TGAGGGGCAGAGAGCGAGACTTC
hsa-miR-424	CAGCAGCAATTCATGTTTTGA
hsa-miR-424	CAGCAGCAATTCATGTTTTGAA
hsa-miR-424	CAGCAGCAATTCATGTTTTGAAA
hsa-miR-424	CAGCAGCAATTCATGTTTTGAAT
hsa-miR-424*	CAAAACGTGAGGCGCTGCTAT
hsa-miR-424*	CAAAACGTGAGGCGCTGCTATA
hsa-miR-424*	CAAAACGTGAGGCGCTGCTA
hsa-miR-424*	CAAAACGTGAGGCGCTGCTAA
hsa-miR-424*	CAAAACGTGAGGCGCTGCTATAA
hsa-miR-424*	CAAAACGTGAGGCGCTGCTATT
hsa-miR-425	AATGACACGATCACTCCCGTTGA
hsa-miR-425	AATGACACGATCACTCCCGTTG
hsa-miR-425	AATGACACGATCACTCCCGTTGT
hsa-miR-425*	ATCGGGAATGTCGTGTCCGCC
hsa-miR-4286	ACCCCACTCCTGGTACCA
hsa-miR-4286	ACCCCACTCCTGGTACC
hsa-miR-4286	ACCCCACTCCTGGTACCAA
hsa-miR-4286	ACCCCACTCCTGGTAC
hsa-miR-4286	ACCCCACTCCTGGTACCAT
hsa-miR-4286	ACCCCACTCCTGGTACCAAA
hsa-miR-4286	ACCCCACTCCTGGTACAA

hsa-miR-4286	ACCCCACTCCTGGTACCATT
hsa-miR-4306	TGGAGAGAAAGGCAGTTCCTGA
hsa-miR-4306	TGGAGAGAAAGGCAGTTCCTGAA
hsa-miR-4306	TGGAGAGAAAGGCAGTTCCTGAT
hsa-miR-4306	TGGAGAGAAAGGCAGTTCCTG
hsa-miR-4306	TGGAGAGAAAGGCAGTTCATGA
hsa-miR-4306	TGGAGAGAAAGGCAGTTCCGGA
hsa-miR-4306	TGGAGAGAAAGGCAGTTCCTGT
hsa-miR-4306	TGGAGAGAAAGGCAGTGCCTGA
hsa-miR-4306	TGGAGAGAAAGGCAGTTACTGA
hsa-miR-4306	TGGAGAGAAAGGCAGTTCCTGAG
hsa-miR-4306	TGGAGAGAAAGGCAGTTCCT
hsa-miR-4306	TGGAGAGAAAGGCAGTTCCTTA
hsa-miR-4306	TGGAGAGAAAGGCAGTTCATGAA
hsa-miR-4306	TGGAGAGAAAGGCAGTTTCTGA
hsa-miR-4306	TGGAGAGAAAGGCAGTTCC
hsa-miR-4306	TGGAGAGAAAGGCAGTTCCGGAA
hsa-miR-4306	TGGAGAGAAAGGCAGTTCCTGTA
hsa-miR-4306	TGGAGAGAAAGGCAGTTCCCGA
hsa-miR-4306	TGGAGAGAAAGGCAGTTACTGAA
hsa-miR-4306	TGGAGAGAAAGGCAGTTCCTTAA
hsa-miR-4306	TGGAGAGAAAGGCAGTGCCTGAA
hsa-miR-4306	TGGAGAGAAAGGCAGTTGCTGA
hsa-miR-4306	TGGAGAGAAAGGCAGTTCAGGA
hsa-miR-4306	TGGAGAGAAAGGCAGTT
hsa-miR-4306	TGGAGAGAAAGGCAGTTCCTGC
hsa-miR-4306	TGGAGAGAAAGGCAGTTCCTGAC
hsa-miR-4306	TGGAGAGAAAGGCAGT
hsa-miR-4306	TGGAGAGAAAGGCAGTTCATGAT
hsa-miR-4306	TGGAGAGAAAGGCAGTCCCTGA
hsa-miR-4306	TGGAGAGAAAGGCAGTTCCTGG
hsa-miR-4306	TGGAGAGAAAGGCAGTTTATGA
hsa-miR-4306	TGGAGAGAAAGGCAGTTCCGGAT
hsa-miR-4306	TGGAGAGAAAGGCAGTTCGTGA
hsa-miR-4306	TGGAGAGAAAGGCAGTGCCTGAT
hsa-miR-4306	TGGAGAGAAAGGCAGTTACTGAT
hsa-miR-4306	TGGAGAGAAAGGCAGTACCTGA
hsa-miR-4306	TGGAGAGAAAGGCAGTTTCTGAA
hsa-miR-4306	TGGAGAGAAAGGCAGTTC
hsa-miR-4306	TGGAGAGAAAGGCAGTTCTTGA
hsa-miR-4306	TGGAGAGAAAGGCAGTTCCTGTT
hsa-miR-4306	TGGAGAGAAAGGCAGTTCCCGAA
hsa-miR-4306	TGGAGAGAAAGGCAGTTGCTGAA
hsa-miR-4306	TGGAGAGAAAGGCAGTTCCTTAT
hsa-miR-4306	TGGAGAGAAAGGCAGTTAATGA
hsa-miR-4306	TGGAGAGAAAGGCAGTTCCTAA
hsa-miR-450a	TTTTGCGATGTGTTCCTAATAT

hsa-miR-450b-5p	TTTTGCAATATGTTCCTGAAT
hsa-miR-454	TAGTGCAATATTGCTTATAGGGT
hsa-miR-454*	ACCCTATCAATATTGTCTCTGT
hsa-miR-484	TCAGGCTCAGTCCCCTCCCGAT
hsa-miR-484	TCAGGCTCAGTCCCCTCCCGA
hsa-miR-484	TCAGGCTCAGTCCCCTCCCGATA
hsa-miR-484	TCAGGCTCAGTCCCCTCCCGAA
hsa-miR-484	TCAGGCTCAGTCCCCTCCCGATT
hsa-miR-484	TCAGGCTCAGTCCCCTCCCGAAA
hsa-miR-486-3p	CGGGGCAGCTCAGTACAGGAT
hsa-miR-486-3p	CGGGGCAGCTCAGTACAGGATA
hsa-miR-486-3p	CGGGGCAGCTCAGTACAGGATT
hsa-miR-486-3p	CGGGGCAGCTCAGTACAGGA
hsa-miR-486-3p	CGGGGCAGCTCAGTACAGGATAA
hsa-miR-486-5p	TCCTGTACTGAGCTGCCCCGAG
hsa-miR-486-5p	TCCTGTACTGAGCTGCCCCGAGA
hsa-miR-486-5p	TCCTGTACTGAGCTGCCCCGAGT
hsa-miR-486-5p	TCCTGTACTGAGCTGCCCCGA
hsa-miR-486-5p	TCCTGTACTGAGCTGCCCCGAA
hsa-miR-486-5p	TCCTGTACTGAGCTGCCCCGAAA
hsa-miR-491-5p	AGTGGGGAACCCTTCCATGAGGA
hsa-miR-497	CAGCAGCACACTGTGGTTTGTA
hsa-miR-497	CAGCAGCACACTGTGGTTTGT
hsa-miR-499-5p	TTAAGACTTGCAGTGATGTTTA
hsa-miR-499-5p	TTAAGACTTGCAGTGATGTTT
hsa-miR-500a*	ATGCACCTGGGCAAGGATTCT
hsa-miR-500a*	ATGCACCTGGGCAAGGATTCTGA
hsa-miR-500a*	ATGCACCTGGGCAAGGATTCTG
hsa-miR-501-3p	AATGCACCCGGGCAAGGATTCT
hsa-miR-501-3p	AATGCACCCGGGCAAGGATTC
hsa-miR-502-3p	AATGCACCTGGGCAAGGATTCA
hsa-miR-502-3p	AATGCACCTGGGCAAGGATTCAG
hsa-miR-502-3p	AATGCACCTGGGCAAGGATTC
hsa-miR-502-3p	AATGCACCTGGGCAAGGATTCT
hsa-miR-503	TAGCAGCGGGAACAGTTCTGCAG
hsa-miR-503	TAGCAGCGGGAACAGTTCTGCA
hsa-miR-503	TAGCAGCGGGAACAGTTCTGCAA
hsa-miR-503	TAGCAGCGGGAACAGTTCTGCAT
hsa-miR-505	CGTCAACACTTGCTGGTTTCCT
hsa-miR-505*	GGGAGCCAGGAAGTATTGATGTT
hsa-miR-505*	GGGAGCCAGGAAGTATTGATGT
hsa-miR-505*	GGGAGCCAGGAAGTATTGATG
hsa-miR-532-3p	CCTCCCACACCCAAGGCTTGCA
hsa-miR-532-3p	CCTCCCACACCCAAGGCTTGCAA
hsa-miR-532-3p	CCTCCCACACCCAAGGCTTGC
hsa-miR-532-5p	CATGCCTTGAGTGTAGGACCGT
hsa-miR-532-5p	CATGCCTTGAGTGTAGGACCGTA

hsa-miR-532-5p	CATGCCTTGAGTGTAGGACCG
hsa-miR-532-5p	CATGCCTTGAGTGTAGGACCGTT
hsa-miR-532-5p	CATGCCTTGAGTGTAGGACCGA
hsa-miR-532-5p	CATGCCTTGAGTGTAGGACCGG
hsa-miR-542-3p	TGTGACAGATTGATAACTGAAA
hsa-miR-542-3p	TGTGACAGATTGATAACTGAA
hsa-miR-542-3p	TGTGACAGATTGATAACTGAAAA
hsa-miR-548a-3p	CAAAACTGGCAATTACTTTTG
hsa-miR-548e	AAAAACTGAGACTACTTTTGCA
hsa-miR-548e	AAAAACTGAGACTACTTTTGCAA
hsa-miR-548e	AAAAACTGAGACTACTTTTGC
hsa-miR-548j	AAAAGTAATTGCGGTCTTTGGT
hsa-miR-548j	AAAAGTAATTGCGGTCTTTGGTA
hsa-miR-548j	AAAAGTAATTGCGGTCTTTGG
hsa-miR-548k	AAAAGTACTTGCGGATTTTGCT
hsa-miR-548l	AAAAGTATTTGCGGGTTTTGT
hsa-miR-548l	AAAAGTATTTGCGGGTTTTGTC
hsa-miR-548n	CAAAAGTAATTGTGGATTTTGT
hsa-miR-548o	CCAAAACTGCAGTTACTTTTG
hsa-miR-548t	CAAAAGTGATCGTGGTTTTTG
hsa-miR-548t	CAAAAGTGATCGTGGTTTTT
hsa-miR-548u	CAAAGACTGCAATTACTTTTGCG
hsa-miR-548w	AAAAGTAACTGCGGTTTTTGCC
hsa-miR-551b	GCGACCCATACTTGGTTTCAGT
hsa-miR-551b	GCGACCCATACTTGGTTTCA
hsa-miR-551b	GCGACCCATACTTGGTTTCAG
hsa-miR-551b*	GAAATCAAGCGTGGGTGAGACCT
hsa-miR-551b*	GAAATCAAGCGTGGGTGAGAC
hsa-miR-551b*	GAAATCAAGCGTGGGTGAGACC
hsa-miR-551b*	GAAATCAAGCGTGGGTGAGACT
hsa-miR-570	CGAAAACAGCAATTACCTTTG
hsa-miR-574-3p	CACGCTCATGCACACACCCACA
hsa-miR-574-3p	CACGCTCATGCACACACCCAC
hsa-miR-574-5p	TGAGTGTGTGTGTGTGAGTGTG
hsa-miR-576-3p	AAGATGTGGAAAAATTGGAAT
hsa-miR-576-3p	AAGATGTGGAAAAATTGGAATC
hsa-miR-576-3p	AAGATGTGGAAAAATTGGAATCC
hsa-miR-576-5p	ATTCTAATTTCTCCACGTCTTT
hsa-miR-576-5p	ATTCTAATTTCTCCACGTCTTTG
hsa-miR-576-5p	ATTCTAATTTCTCCACGTCTT
hsa-miR-582-3p	TAACTGGTTGAACAACTGAAC
hsa-miR-582-5p	TTACAGTTGTTCAACCAGTTACT
hsa-miR-589	TGAGAACCACGTCTGCTCTGA
hsa-miR-589	TGAGAACCACGTCTGCTCTGAAA
hsa-miR-589	TGAGAACCACGTCTGCTCTGAG
hsa-miR-590-3p	TAATTTTATGTATAAGCTAGT
hsa-miR-590-3p	TAATTTTATGTATAAGCTAGTC

hsa-miR-590-3p	TAATTTTATGTATAAGCTAGTT
hsa-miR-590-5p	GAGCTTATTCATAAAAGTGCAG
hsa-miR-590-5p	GAGCTTATTCATAAAAGTGCA
hsa-miR-598	TACGTCATCGTTGTCATCGTCA
hsa-miR-598	TACGTCATCGTTGTCATCGTC
hsa-miR-598	TACGTCATCGTTGTCATCGTCAT
hsa-miR-598	TACGTCATCGTTGTCATCGTCAA
hsa-miR-598	TACGTCATCGTTGTCATCGTCT
hsa-miR-618	AAACTCTACTTGTCCTTCTGAGT
hsa-miR-618	AAACTCTACTTGTCCTTCTGAG
hsa-miR-625	AGGGGGAAAGTTCTATAGTC
hsa-miR-625	AGGGGGAAAGTTCTATAGTCCT
hsa-miR-625	AGGGGGAAAGTTCTATAGTCC
hsa-miR-625	AGGGGGAAAGTTCTATAGTCA
hsa-miR-625	AGGGGGAAAGTTCTATAGTCCTT
hsa-miR-625	AGGGGGAAAGTTCTATAGTCCTA
hsa-miR-625	AGGGGGAAAGTTCTATAGTCCTG
hsa-miR-625	AGGGGGAAAGTTCTATAGTCT
hsa-miR-625*	GACTATAGAACTTTCCCCCTCA
hsa-miR-625*	GACTATAGAACTTTCCCCCTCAA
hsa-miR-625*	GACTATAGAACTTTCCCCCTC
hsa-miR-625*	GACTATAGAACTTTCCCCCTCT
hsa-miR-625*	GACTATAGAACTTTCCCCCTCAT
hsa-miR-625*	GACTATAGAACTTTCCCCCTCC
hsa-miR-628-5p	ATGCTGACATATTTACTAGAGG
hsa-miR-629	TGGGTTTACGTTGGGAGAACT
hsa-miR-629	TGGGTTTACGTTGGGAGAAC
hsa-miR-629	TGGGTTTACGTTGGGAGAACTT
hsa-miR-629	TGGGTTTACGTTGGGAGAACTTA
hsa-miR-629*	GTTCTCCCAACGTAAGCCCAGC
hsa-miR-629*	GTTCTCCCAACGTAAGCCCAG
hsa-miR-629*	GTTCTCCCAACGTAAGCCCAGT
hsa-miR-641	AAAGACATAGGATAGAGTCACCT
hsa-miR-642a	GTCCCTCTCCAAATGTGTCTTG
hsa-miR-651	TTTAGGATAAGCTTGACTTTTG
hsa-miR-651	TTTAGGATAAGCTTGACTTTT
hsa-miR-651	TTTAGGATAAGCTTGACTTTTGT
hsa-miR-652	AATGGCGCCACTAGGGTTGTGT
hsa-miR-652	AATGGCGCCACTAGGGTTGT
hsa-miR-652	AATGGCGCCACTAGGGTTGTG
hsa-miR-652	AATGGCGCCACTAGGGTTGTGA
hsa-miR-652	AATGGCGCCACTAGGGTTGTGC
hsa-miR-652	AATGGCGCCACTAGGGTTGTGTT
hsa-miR-660	TACCCATTGCATATCGGAGTTG
hsa-miR-660	TACCCATTGCATATCGGAGTTGT
hsa-miR-660	TACCCATTGCATATCGGAGTT
hsa-miR-664	TATTCATTTATCCCCAGCCTACA

hsa-miR-664	TATTCATTTATCCCCAGCCTAC
hsa-miR-664*	ACTGGCTAGGGAAAATGATTGGA
hsa-miR-671-5p	AGGAAGCCCTGGAGGGGCTGGAG
hsa-miR-7	TGGAAGACTAGTGATTTTGTTGT
hsa-miR-7	TGGAAGACTAGTGATTTTGTTG
hsa-miR-7-1*	CAACAAATCACAGTCTGCCATA
hsa-miR-7-1*	CAACAAATCACAGTCTGCCAT
hsa-miR-7-1*	CAACAAATCACAGTCTGCCATT
hsa-miR-7-1*	CAACAAATCACAGTCTGCCATAA
hsa-miR-7-1*	CAACAAATCACAGTCTGCCATAT
hsa-miR-720	TCTCGCTGGGGCCTCCA
hsa-miR-744	TGCGGGGCTAGGGCTAACAGCA
hsa-miR-744	TGCGGGGCTAGGGCTAACAGCAA
hsa-miR-744	TGCGGGGCTAGGGCTAACAGCAT
hsa-miR-744	TGCGGGGCTAGGGCTAACAGC
hsa-miR-744	TGCGGGGCTAGGGCTAACAGCAG
hsa-miR-760	CGGCTCTGGGTCTGTGGGGAGT
hsa-miR-760	CGGCTCTGGGTCTGTGGGGAG
hsa-miR-760	CGGCTCTGGGTCTGTGGGGAGTT
hsa-miR-760	CGGCTCTGGGTCTGTGGGGA
hsa-miR-766	ACTCCAGCCCCACAGCCTCAG
hsa-miR-766	ACTCCAGCCCCACAGCCTCAGC
hsa-miR-766	ACTCCAGCCCCACAGCCTCAGA
hsa-miR-766	ACTCCAGCCCCACAGCCTCAGT
hsa-miR-766	ACTCCAGCCCCACAGCCTCAGCA
hsa-miR-769-3p	CTGGGATCTCCGGGGTCTTGGTT
hsa-miR-769-5p	TGAGACCTCTGGGTTCTGAGCT
hsa-miR-769-5p	TGAGACCTCTGGGTTCTGAGC
hsa-miR-769-5p	TGAGACCTCTGGGTTCTGAGCTA
hsa-miR-874	CTGCCCTGGCCCGAGGGACCGA
hsa-miR-874	CTGCCCTGGCCCGAGGGACCGAC
hsa-miR-874	CTGCCCTGGCCCGAGGGACCGAT
hsa-miR-874	CTGCCCTGGCCCGAGGGACCGAA
hsa-miR-877	GTAGAGGAGATGGCGCAGGGGAC
hsa-miR-877	GTAGAGGAGATGGCGCAGGGGAA
hsa-miR-877	GTAGAGGAGATGGCGCAGGGGA
hsa-miR-877	GTAGAGGAGATGGCGCAGGG
hsa-miR-9	TCTTTGGTTATCTAGCTGTATGA
hsa-miR-9	TCTTTGGTTATCTAGCTGTATGT
hsa-miR-9*	ATAAAGCTAGATAACCGAAAGT
hsa-miR-9*	ATAAAGCTAGATAACCGAAAGTA
hsa-miR-92a	TATTGCACTTGTCCCGGCCTGT
hsa-miR-92a	TATTGCACTTGTCCCGGCCTGTA
hsa-miR-92a	TATTGCACTTGTCCCGGCCTG
hsa-miR-92a	TATTGCACTTGTCCCGGCCTGTT
hsa-miR-92a	TATTGCACTTGTCCCGGCCTGA
hsa-miR-92a	TATTGCACTTGTCCCGGCCTGAA

hsa-miR-92a	TATTGCACTTGTCCCGGCCTGG
hsa-miR-92a	TATTGCACTTGTCCCGGCCTGC
hsa-miR-92a	TATTGCACTTGTCCCGGCCTGAT
hsa-miR-92a	TATTGCACTTGTCCCGGCCTGGA
hsa-miR-92a	TATTGCACTTGTCCCGGCCTGCA
hsa-miR-92a	TATTGCACTTGTCCCGGCCTGTG
hsa-miR-92a	TATTGCACTTGTCCCGGCCTGTC
hsa-miR-92a-1*	AGGTTGGGATCGGTTGCAATGCT
hsa-miR-92a-1*	AGGTTGGGATCGGTTGCAATGC
hsa-miR-92a-1*	AGGTTGGGATCGGTTGCAATGCG
hsa-miR-92a-1*	AGGTTGGGATCGGTTGCAATGCA
hsa-miR-92a-1*	AGGTTGGGATCGGTTGCAATGCC
hsa-miR-92a-2*	GGGTGGGGATTTGTTGCATTACT
hsa-miR-92a-2*	GGGTGGGGATTTGTTGCATTAC
hsa-miR-92b	TATTGCACTCGTCCCGGCCTC
hsa-miR-92b	TATTGCACTCGTCCCGGCCTCC
hsa-miR-92b	TATTGCACTCGTCCCGGCCTCT
hsa-miR-92b	TATTGCACTCGTCCCGGCCTCA
hsa-miR-92b	TATTGCACTCGTCCCGGCCTCCT
hsa-miR-92b	TATTGCACTCGTCCCGGCCTCCA
hsa-miR-92b	TATTGCACTCGTCCCGGCCTCAA
hsa-miR-92b*	AGGGACGGGACGCGGTGCAGTGT
hsa-miR-92b*	AGGGACGGGACGCGGTGCAGTG
hsa-miR-92b*	AGGGACGGGACGCGGTGCAGT
hsa-miR-92b*	AGGGACGGGACGCGGTGCAGTGA
hsa-miR-93	CAAAGTGCTGTTCGTGCAGGTAG
hsa-miR-93	CAAAGTGCTGTTCGTGCAGGTA
hsa-miR-93	CAAAGTGCTGTTCGTGCAGGTAA
hsa-miR-93	CAAAGTGCTGTTCGTGCAGGTAT
hsa-miR-93*	ACTGCTGAGCTAGCACTTCCCGA
hsa-miR-93*	ACTGCTGAGCTAGCACTTCCCG
hsa-miR-940	AAGGCAGGGCCCCCGCTCCCCT
hsa-miR-941	CACCCGGCTGTGTGCACATGTGC
hsa-miR-941	CACCCGGCTGTGTGCACATGTG
hsa-miR-941	CACCCGGCTGTGTGCACATGTGT
hsa-miR-941	CACCCGGCTGTGTGCACATGTGA
hsa-miR-942	TCTTCTCTGTTTTGGCCATGTG
hsa-miR-942	TCTTCTCTGTTTTGGCCATGT
hsa-miR-942	TCTTCTCTGTTTTGGCCATGTGA
hsa-miR-944	AAATTATTGTACATCGGATGAG
hsa-miR-944	AAATTATTGTACATCGGATGAGA
hsa-miR-944	AAATTATTGTACATCGGATGA
hsa-miR-944	AAATTATTGTACATCGGATGAGT
hsa-miR-95	TTCAACGGGTATTTATTGAGCA
hsa-miR-98	TGAGGTAGTAAGTTGTATTGTT
hsa-miR-98	TGAGGTAGTAAGTTGTATTGT
hsa-miR-98	TGAGGTAGTAAGTTGTATTGTTA

hsa-miR-98	TGAGGTAGTAAGTTGTATTGTTT
hsa-miR-98	TGAGGTAGTAAGTTGTATTGTA
hsa-miR-98	TGAGGTAGTAAGTTGTATTGTTG
hsa-miR-99a	AACCCGTAGATCCGATCTTGT
hsa-miR-99a	AACCCGTAGATCCGATCTTGTGA
hsa-miR-99a	AACCCGTAGATCCGATCTTGTGT
hsa-miR-99b	CACCCGTAGAACCGACCTTGCG
hsa-miR-99b	CACCCGTAGAACCGACCTTGCGA
hsa-miR-99b	CACCCGTAGAACCGACCTTGC
hsa-miR-99b	CACCCGTAGAACCGACCTTGCGT
