# Supplementary material for: Comprehensive microRNA profiling in B-cells of human centenarians by massively parallel sequencing
Source: BMC Genomics. 2012 Jul 31;13:353. doi: 10.1186/1471-2164-13-353 (PMC3563618; doi:10.1186/1471-2164-13-353)
Supplement: Additional file 4 — Table S4. Putative and Confirmed Targets of differentially expressed miRNAs. [file 1471-2164-13-353-S4.pdf]

Table S4: Putative and Confirmed Targets of differentially expressed miRNAs

miRNA	Targets
hsa-mIR-122	GIT1, CLIC4, GYS1, FKBP5, SOX11, CYCLING1*, BCL-W*, ADAM17*, SLC7A1*, AKT3*, NUMBL*, FOXJ3*, XPO6*, AP3M2*, G6PC3*, GALNT10*, RIPK5*, TPD52L2*, FUNDC2*, MAPK11*, ALS2CR13*, BACH2*, ATP11A*, ATP1A2*, DUSP2*, OSMR*, RABIF*, PALM*, AACS*, TBX19*, UBAP2*, EGLN3*, NCAM1*, MeCP2*, CS*, FOXP1*, RAB11FIP1*, RAB6B*, TRIB1*, TTYH3*, ALDOA*, ANXA11*, CLDN18*, ENTPD4*, NFATC2IP*, ANK2*, MEP1A*, NFATC1*, SLC7A11*
hsa-mIR-20b	MYT1L, BCL11B, CAMTA1, FAM102A, ZBTB7A, ANKRD52, KIAA0494, EPHA4, ARHGAP12, GLIS3, ZC3H12C, AKAP11, C11orf30, FBXW11, NR4A3, PRRG1, ZC3H7B, ZNFX1, LDLR, RUNX3, SLC46A3, FAT2, XRRA1, PTPRD, M6PR, ITGB8, VLDLR, PKD2, MKRN1, CHD9, RAB5B, SR140, EZH1, TBL1X, CLIP4, PURB, STAT3, NAPEPLD, PFN2, TET3, MAP3K14, KIAA0513, MTMR3, KLHL28, MFAP3L, USP31, GPR6, ZBTB4, UBFD1, FCHO2, ATXN1, FRS2, E2F1, PKNOX1, TP53INP1, NBEA, TBC1D20, KLF12, ARID4A, CCND1, SLITRK3, CDKN1A, NPAS2, GPR137C, PDGFRA, UNK, OTUD4, ZFYVE26, SLC40A1, BTBD7, PLEKHA3, DPYSL5, VANGL1, BCL2L2, ZBTB41, NTRK2, RBL2, MKL2, TXNIP, NCOA3, ZFP91, KPNA3, ANKIB1, ENPP5, C5orf41, RPS6KA2, WDR37, NPAT, TXLNA, MAP3K2, BMPR2, SERP1, C2CD2, CRAMP1L, ANKFY1, RUFY2, FURIN, ZNF512B, ANKRD29, TBC1D9, FYCO1, ESR1*
hsa-mIR-454	C10orf140, RNF38, LDLR, PTEN, TBL1XR1, MIER1, C7orf60, MYBL1, TSC1, SBF2, DDX6, FBXO28, WNT1, SULF1, DICER1, PIGA, ACSL4, RNF41, PAN3, MAF, DENND4C, CPEB4, EREG, CREB5, MBNL1, CPEB2, ACVR1, MYT1L, MXD1, ZFPM2, EPS15, C1orf144, WDR20, PGM2L1, SOS2, BACH2, ZBTB4, ARL6IP1, HBP1, EPC2, CPEB1, MLLT6, EIF2C1, MTMR9, NPTX1, CUL3, PIK3C2A, IGF1, SHANK2, FAM104A, ENPP5, EIF2C4, BRWD1, FAM178A, CHST1, ZNF217, JARID2
hsa-mIR-99b	BAZ2A
hsa-mIR-363	CPEB3, SLC12A5, RBM47, PIK3R3, CD69, EIF4G2, SH3PXD2A, CSMD3, PCDH11X, NFIB, FNDC3B, RNF38, GRIA1, BSN, BCL2L11, MYO1B, RAB23, B3GALT2, TEAD1, NSF, RAP1B, NFIX, MTF1, FBXW7, MSRB3, CPEB4, GIT2, SYN2, CPEB2, IRS2, CSMD1, LIN54, MAP2K4, REV3L, ANKRD13C, MARK1, TULP4, BTG2, PMEPA1, PITPNM2, LHFPL2, TTC9, ADAM10, MEF2D, KLHL14, ARMC1, SERTAD2, PCDH7, KIAA0831, FNIP1, GATAD2B, RAG1, MAN2A1, SLC6A1
hsa-mIR-148a	MTF1, C1orf144, MTMR9, MITF, WNT1, SERBP1, MGAT4A, ATP11A, TGIF2, BCL2L11, SYNJ1, C18orf25, MLL, KIAA0232, CPEB4, ZDHHC7, ATP6AP2, MXD1, EPS15, MIER1, PTEN, NPTN, RICTOR, PPP1R10, MEOX2, ITGA5, B4GALT5, OSBPL11, TXNIP, C5orf30, SNN, SLC24A3, PXR*, DNMT3B1*, DNMT3B3*, DNMT-1*
hsa-mIR-181	DDX3X, TNPO1, FOXP1, CPEB4, ZNF440, TRIM2, FIGN, ATP2B1, HSPC159, SLAIN2, ATP2B2, DLGAP2, GIGYF1, BTBD3, ADAM11, KIAA0528, LARP4, CTDSPL, ZNF594, PITPNB, MBOAT2, OSBPL3, EPB41, PLDN, MLL3, ROD1, CLASP1, HIC2, KRAS, ITGB8, ITSN1, TIMP3, RAP1B, BRWD1, BCL2, KPNB1, SSX2IP, ZADH2, BCLAF1, GPD1L, TMEM132D, SENP1, PDE5A, PHACTR4, PCDHA13, PCDHA11, TMCC1, ENTPD6, ZBTB4, PCDHA3, ZFP14, KIF3B, ATXN1, DDX3Y, PAPD5, PCDHA1, PLAG1, PPP1R3B, PKNOX2, SS18L1, PCDHA5, DERL1, PCDHAC1, SPRY4, HOXC8, SLC2A3, PDGFRA, CUL3, ST8SIA4, KPNA4, SIPA1L2, KIAA2022, KIAA0195, CBX7, PCDHA2, C7orf41, FBXO34, ARSJ, C5orf41, TGFBRAP1, PCDHA6, GNB4, ADO, PCDHAC2, ADAMTS6, PCDHA8, SLITRK1, C11orf41, SERTAD2, RCOR1, NPTXR, BIRC6, RLF, PCDHA12, ADCY9, NOVA1, LIN28B, PCDHA10, CTTNBP2NL, PCDHA4, IPO5, PCDHA7, MAPK1

hsa-mIR-192	PABPC4, DHFR*
hsa-mIR-361-5p	SP1, RPA1
hsa-mIR-9	HIC2, LDLRAP1, ZBTB41, SHROOM4, PRDM1, MAP3K3, SCUBE3, ALCAM, TNC, LSM14A, BAHD1, ROD1, SLC12A5, HN1L, CPEB3, PCGF5, FOXP1, FRMD6, CPEB4, CPEB2, GABRB2, FBN1, AP1S2, PI4K2A, ARFGEF2, PHLDB2, PHF20L1, SMARCE1, NOX4, LZTS2, ZBTB39, SPTLC2, SHC1, MMP16, TMTC1, SMURF1, TNKS, KIAA0240, CEP350, COG3, RAG1AP1, FOXP4, GOT1, RAB34, KCNJ2, LPHN1, SOCS5, CSDA, ZKSCAN1, MDGA1, MKL2, FIGNL2, STARD13, UBE3C, SLC31A2, MTHFD2, SFXN2, PRRX1, MEGF10, ONECUT1, ELAVL1, LIN28B, DIAPH2, trkC*, BACE1*, PRDM1*, REST*, NFKB1*, FOXO1*, CDH1*

***Experimentally Confirmed Targets in Literature and reported by miRecords. [1-4]**

1. Braconi C, Huang N, Patel T: **MicroRNA-dependent regulation of DNA methyltransferase-1 and tumor suppressor gene expression by interleukin-6 in human malignant cholangiocytes**. *Hepatology* 2010, **51**(3):881-890.
2. Tili E, Michaille JJ, Cimino A, Costinean S, Dumitru CD, Adair B, Fabbri M, Alder H, Liu CG, Calin GA *et al*: **Modulation of miR-155 and miR-125b levels following lipopolysaccharide/TNF-alpha stimulation and their possible roles in regulating the response to endotoxin shock**. *J Immunol* 2007, **179**(8):5082-5089.
3. Tsai WC, Hsu PW, Lai TC, Chau GY, Lin CW, Chen CM, Lin CD, Liao YL, Wang JL, Chau YP *et al*: **MicroRNA-122, a tumor suppressor microRNA that regulates intrahepatic metastasis of hepatocellular carcinoma**. *Hepatology* 2009, **49**(5):1571-1582.
4. Xiao F, Zuo Z, Cai G, Kang S, Gao X, Li T: **miRecords: an integrated resource for microRNA-target interactions**. *Nucleic Acids Research* 2009, **37**:D105-D110.
