# Supplementary material for: Comprehensive microRNA profiling in B-cells of human centenarians by massively parallel sequencing
Source: BMC Genomics. 2012 Jul 31;13:353. doi: 10.1186/1471-2164-13-353 (PMC3563618; doi:10.1186/1471-2164-13-353)
Supplement: Additional file 5 — Table S5. Significantly over-represented GO categories of predicted targets of miRNA differentially expressed in B-Cells of Centenarians as compared to Controls. [file 1471-2164-13-353-S5.pdf]

Supplementary Table S5: Significantly over-represented GO categories of predicted targets of miRNA differentially expressed in B-Cells of Centenarians as compared to Controls

GO TERM	GO ID	Genes Found	Total Genes in Set	p-Value
nervous system development	GO:0007399	39	716	3.92E-28
developmental process	GO:0032502	94	3347	1.16E-23
multicellular organismal development#system development	GO:0048731	59	1605	1.16E-23
anatomical structure development	GO:0048856	67	2005	5.65E-23
cellular process#regulation of cellular process	GO:0050794	131	5704	2.19E-22
multicellular organismal development	GO:0007275	72	2299	3.79E-22
regulation of biological process	GO:0050789	137	6140	3.79E-22
biological regulation	GO:0065007	145	6731	9.00E-22
metabolic process#regulation of metabolic process	GO:0019222	99	4150	1.04E-17
negative regulation of cellular process	GO:0048523	42	1137	8.15E-17
regulation of cellular metabolic process	GO:0031323	94	3933	8.15E-17
gene expression#regulation of gene expression	GO:0010468	91	3833	5.92E-16
negative regulation of biological process	GO:0048519	42	1182	8.95E-16
multicellular organismal process	GO:0032501	89	3822	7.24E-15
regulation of nucleobase, nucleoside, nucleotide and nucleic acid metabolic process	GO:0019219	85	3713	1.66E-13
regulation of transcription, DNA-dependent	GO:0006355	79	3358	2.50E-13
transcription, DNA-dependent	GO:0006351	80	3439	3.61E-13
RNA biosynthetic process	GO:0032774	80	3444	3.82E-13
RNA metabolic process	GO:0016070	91	4155	4.13E-13
regulation of transcription	GO:0045449	83	3654	5.42E-13
biopolymer metabolic process	GO:0043283	142	7940	2.64E-12
transcription	GO:0006350	84	3803	2.86E-12
cellular developmental process	GO:0048869	47	1810	1.52E-09
cell differentiation	GO:0030154	47	1810	1.52E-09
homophilic cell adhesion	GO:0007156	17	206	2.93E-09
nucleobase, nucleoside, nucleotide and nucleic acid metabolic process	GO:0006139	106	5848	6.69E-09
positive regulation of biological process	GO:0048518	31	1062	7.54E-08
gene expression	GO:0010467	98	5454	7.57E-08
macromolecule metabolic process	GO:0043170	167	11144	1.77E-07
cell-cell adhesion	GO:0016337	19	347	1.91E-07
cellular component organization and biogenesis	GO:0016043	66	3277	3.36E-07
positive regulation of cellular process	GO:0048522	28	954	3.68E-07
enzyme linked receptor protein signaling pathway	GO:0007167	17	300	6.84E-07
transcription from RNA polymerase II promoter	GO:0006366	21	640	1.46E-06
primary metabolic process	GO:0044238	181	12764	2.40E-06
biological adhesion	GO:0022610	27	960	2.51E-06
cell adhesion	GO:0007155	27	960	2.51E-06
negative regulation of cellular metabolic process	GO:0031324	18	381	3.60E-06
cellular metabolic process	GO:0044237	179	12668	3.82E-06
negative regulation of metabolic process	GO:0009892	19	436	5.31E-06
cell-cell signaling	GO:0007267	20	640	1.05E-05
neurological system process#transmission of nerve impulse	GO:0019226	16	330	1.17E-05
cell differentiation#cell development	GO:0048468	31	1242	1.20E-05
cell cycle process	GO:0022402	19	625	4.06E-05
transmembrane receptor protein tyrosine kinase signaling pathway	GO:0007169	12	210	5.73E-05
neurological system process#transmission of nerve impulse#synaptic transmission	GO:0007268	14	290	5.94E-05
negative regulation of transcription	GO:0016481	14	291	6.06E-05
cell proliferation	GO:0008283	21	745	6.66E-05
regulation of signal transduction	GO:0009966	22	800	6.76E-05
multicellular organismal development#system development#organ development	GO:0048513	28	1141	6.76E-05
cell communication	GO:0007154	90	5560	7.68E-05
negative regulation of nucleobase, nucleoside, nucleotide and nucleic acid metabolic process	GO:0045934	14	319	0.000154
nervous system development#central nervous system development	GO:0007417	12	235	0.000154
negative regulation of transcription, DNA-dependent	GO:0045892	11	203	0.000209
intracellular signaling cascade	GO:0007242	40	1965	0.000216
protein kinase cascade	GO:0007243	15	376	0.000216
anatomical structure morphogenesis	GO:0009653	25	1047	0.000437
regulation of progression through cell cycle	GO:0000074	14	353	0.000437
negative regulation of progression through cell cycle	GO:0045786	11	225	0.000499
regulation of cell cycle	GO:0051726	14	359	0.000509
striated muscle development	GO:0014706	7	88	0.000705
multicellular organismal development#embryonic development	GO:0009790	11	235	0.000707
cell cycle	GO:0007049	21	839	0.000802
